# Brain Region-dependent Heterogeneity and Dose-dependent Difference in Transient Microglia Population Increase during Lipopolysaccharide-induced Inflammation

**DOI:** 10.1038/s41598-018-20643-3

**Published:** 2018-02-02

**Authors:** Eriko Furube, Shintaro Kawai, Haruna Inagaki, Shohei Takagi, Seiji Miyata

**Affiliations:** 1 Department of Applied Biology, Kyoto, 606-8585 Japan; 2 The Center for Advanced Insect Research Promotion, Kyoto Institute of Technology, Matsugasaki, Sakyo-ku, Kyoto, 606-8585 Japan

**Keywords:** Glial biology, Neuroimmunology

## Abstract

Numerous studies have reported the importance of microglial activation in various pathological conditions, whereas little attention has been given to the point for dynamics of microglial population under infection-induced inflammation. In the present study, the single systemic stimulation of 100 μg/kg lipopolysaccharide (LPS) induced robust microglial proliferation only in the circumventricular organs (CVOs) and their neighboring brain regions. More than half of microglia similarly showed proliferative activity in the CVOs and their neighboring brain regions after 1 mg/kg LPS stimulation, while this stimulation expanded microglia-proliferating brain regions including the hypothalamus, medulla oblongata, and limbic system. Microglia proliferation resulted in a transient increase of microglial density, since their density almost returned to basal levels within 3 weeks. Divided microglia survived at the same rate as non-divided ones. Proliferating microglia frequently expressed a resident microglia marker Tmem119, indicating that increase of microglia density is due to the proliferation of resident microglia. Thus, the present study demonstrates that transient increase in microglia density depends on the brain region and dose of LPS during infection-induced inflammation and could provide a new insight on microglia functions in inflammation and pathogenesis of brain diseases.

## Introduction

Microglia are innate immune cells in the brain that are diffusely distributed throughout the parenchyma and function in brain immune defenses. The microglial population in the adult rodent brain accounts for 5 to 12% of the total number of cells^1^. In the human brain, microglia account for 0.5 to 16.6% of the total population of brain cells and show similar regional variability to that reported in rodents^2^. Microglia have the ability to respond to many types of brain homeostatic disturbances under pathological brain conditions and are rapidly transformed from a ramified to amoeboid morphology, namely “activated microglia”^3–5^. Ramified microglia, composed of long branching processes and a small cellular body, function as surveying cells by actively sensing the surrounding microenvironment via dynamic fine cellular processes^4^. Activated amoeboid microglia are hypertrophic, typically have a less dendritic shape, and participate in many functions including phagocytosis and cytokine release^6^.

Microglial proliferation in the adult rodent brains is slow with increases at a rate of only a few percent per week under physiologically healthy conditions^1,7,8^. In the mouse and human brain, the microglial density remains remarkably stable, but microglia turnover several times during a lifetime^9^. They further have shown that microglia turnover is maintained by coupled proliferation and apoptosis of resident microglia rather than the infiltration of bone marrow-derived immune cells^9^. However, microglia increase their population by both proliferation of the resident microglia and recruitment of bone marrow-derived immune cells under pathological brain conditions: traumatic and ischemic brain injuries, Alzheimer’s disease, prion diseases, and multiple sclerosis^3–5,10^. The mice deficient for fractalkine receptor that is involved in the adhesion and migration of microglia and other immune cells reveal lower brain levels of amyloid-β and amyloid deposits in an Alzheimer’s model mouse^11^. This is due to an overall greater phagocytic capacity by higher proliferative activity of microglia and subsequent increase of their number around individual plaques. The inhibition of microglial proliferation by inhibitors against colony stimulating factor receptor 1 (CSF1R) has been shown to attenuate the neuronal damage associated with prion diseases^5,12^ and Alzheimer disease^13^. Thus, the proliferation of microglia is an important disease-associated event under pathological brain conditions as well as the activation of microglia.

Lipopolysaccharide (LPS), a component of the Gram-negative bacterial cell wall, is the most commonly used and established inducer for the experimental inflammation model of animals. The absence of microglial proliferation in the cerebral cortex (Cx) is reported even after the consecutive four-day intraperitoneal administration of 1 mg/kg LPS^14^. The percentage of microglial proliferation is found to be 10.1% per week after the intraperitoneal administration of 1 mg/kg LPS every other day, when proliferation analysis is performed in whole brains using ^2^H_2_O labeling^7^. Recently, we have demonstrated that the single intraperitoneal administration of 1 mg/kg LPS induces marked increases in microglial proliferation in the circumventricular organ (CVOs), including the organum of vasculosum of the lamina terminalis (OVLT), subfornical organ (SFO), and area postrema (AP) in the adult mouse brain^15^. The CVOs are brain regions that do not express endothelial tight junction proteins^16^, lack the typical blood-brain barrier (BBB), and show relatively high vascular permeability^17,18^. This indicates that the CVOs act as the main entry route of blood-derived inflammatory cytokines and pathogens, and convey their information into inflammatory and thermoregulatory brains regions^19–21^. The levels of LPS in the blood stream is significantly increased after peripheral administration of low dose LPS, but brain uptake of circulating LPS is so little that most effects of peripherally administered LPS are probably mediated through TLR4 located outside the BBB^22,23^. Recently, however, circulating LPS is shown to be incorporated into ependymal and endothelial cells in the CVOs by plasma-lipoprotein mediated transport^24^. The expression levels of Toll-like receptor 4 (TLR4) and its co-receptor CD14 are found to be markedly higher in the sensory CVOs than in other brain regions^25–27^. The peripheral and central administration of LPS is shown to stimulate TLR4 on astrocytes and ependymal cells in the sensory CVOs, which leads to faster proinflammatory responses such as the activation of JAK-signal transducer and activator of transcription 3 (STAT3) and nuclear factor-κ B (NF-κ B) in the CVOs than that in other brain regions^27–29^. Thus, the CVOs are the primary brain structures that rapidly respond to proinflammatory stimuli present in the bloodstream including LPS and cytokines.

A large number of evidences indicate that there is a causative relationship between infection-induced brain inflammation and depression in the adult human^30^. Moreover, proinflammatory cytokines are shown to be elevated in the plasma or cerebrospinal fluid of depressed patients, which are associated with the severity of depression^31^. In animal models, stimulation that promote the production of proinflammatory cytokines are shown to result in depressive-like behavior. Inflammation-dependent activation of microglia produces proinflammatory cytokines and secondary messengers and thereby elicit a sickness behavior syndrome^32^. Low-level neuroinflammation by systemic LPS robustly impairs retrieval of previously learned context discrimination together with the reduction of orthogonalization of neuronal ensembles in the hippocampus^33^. The systemic administration of low dose of LPS caused transient neuroprotection against ischemia and stroke in brains^34^. Conversely, systemic inflammation has significant exacerbation effects on the progression of neurodegenerative disease by inducing greater increases in proinflammatory cytokines and cell death^35,36^. Thus, chronic activation of microglia is intimately associated with the pathogenesis of a variety of brain diseases, although sickness behavior is adaptive and protective responses in normal healthy animals after acute inflammatory stimulation.

Thus, infection-induced microglia dynamics are important in the pathogenesis of many brain diseases, while evidences for microglial proliferation during such inflammatory brain conditions remain wholly insufficient. Therefore, we herein attempted to elucidate the proliferation of resident microglia in mouse brain during LPS-induced inflammation: (1) brain region-dependent heterogeneity; whether LPS-induced microglial proliferation is limited to the CVOs or occurs in other brain regions; (2) dose-dependent difference; whether microglial proliferation is induced by LPS stimulation at the dose of 100 μg/kg as well as 1 mg/kg; (3) endogenous microglia proliferation; whether microglia density is increased by the proliferation of brain-resident microglia or the infiltration of circulating immune cells; (4) fate of proliferated microglia; how long increased density of microglia is maintained and whether divided microglia survive or die. The present study is the first demonstration for brain region-dependent heterogeneity of transient and robust microglia proliferation during infection-induced inflammation. Moreover, such microglia proliferation is induced by low dose of LPS, indicating that a transient increase in microglia population could occur during mild inflammatory stimulation.

## Results

**Figure 1:**
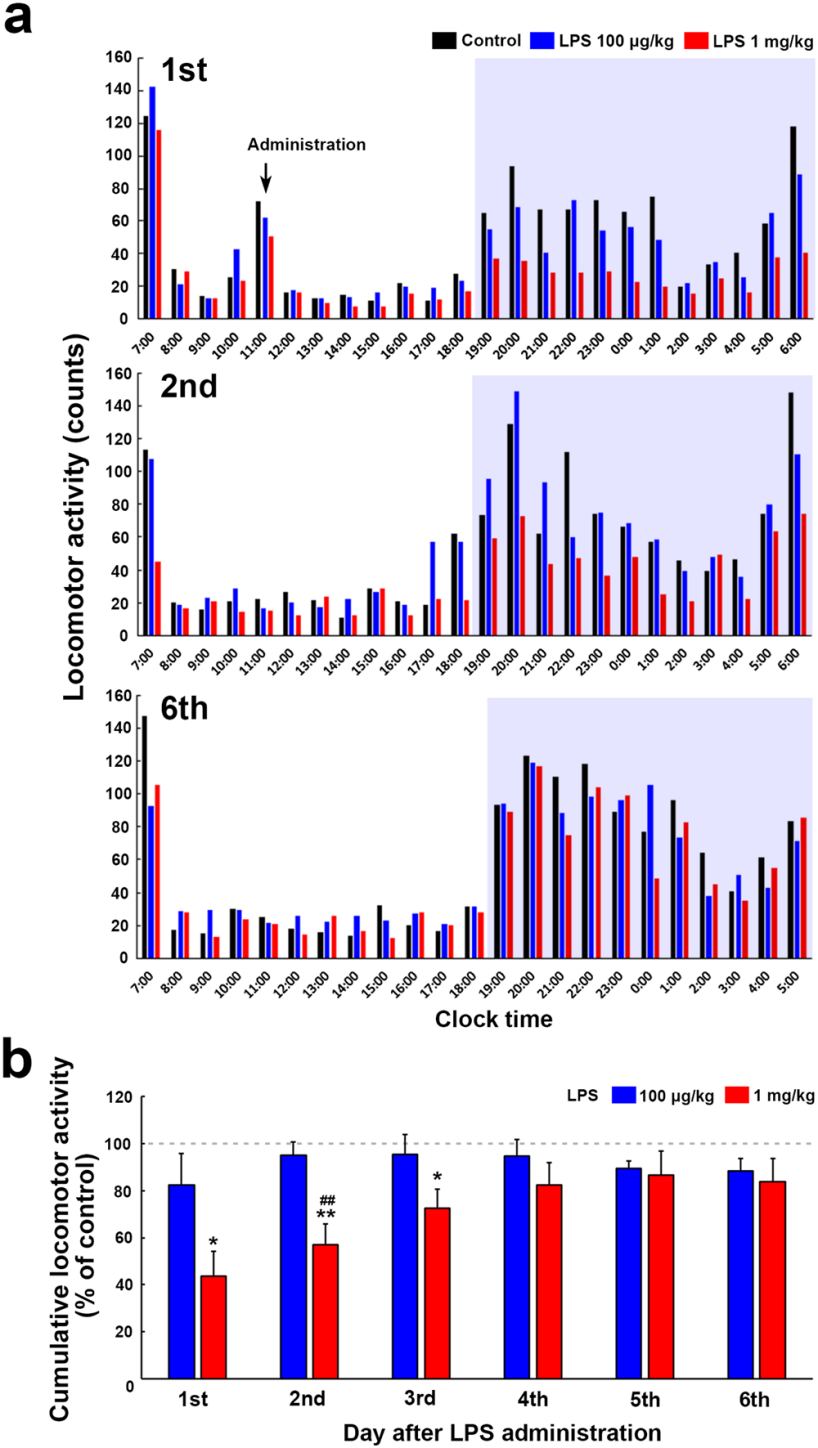
Changes in the locomotor activity of mice after the single administration of *Escherichia coli* (serotype 055:B5) LPS. Mice received the single intraperitoneal administration of 100 μg/kg LPS, 1 mg/kg LPS, or saline on 11:30 and mean counts of the locomotor activity was monitored over 10 min intervals by E-mitter mouse telemetry system. Mean locomotor activity calculated over 60 min intervals during the daytime was not changed among control and LPS-treated animal groups (**a**). The administration of 1 mg/kg LPS reduced mean locomotor activity during the nighttime on the first and second day of 1 mg/kg LPS administration, but it returned to control level on the sixth day. Mean locomotor activity during the nighttime was likely to be lower on the first day of 100 μg/kg LPS administration. The cumulative values of the locomotor activity during nighttime was significantly lower from the first to third day upon the single administration of 1 mg/kg LPS, although no significant changes in the cumulative locomotor activity was observed when mice received 100 μg/kg LPS administration (**b**). Data were expressed as the mean (±SE) of 8~10 animals. *P < 0.05, **P < 0.01 vs the control and ^##^P < 0.01 between 100 μg/kg and 1 mg/kg LPS by ANOVA with Tukey post hoc test.

The intraperitoneal administration of low dose of LPS has been reported to induce hyperthermia in mice, whereas that of high dose of LPS causes hypothermia^37^. To determine dose-dependent difference of sickness behavior, the locomotor activity of mice was monitored by an E-Mitter mouse telemetory system. Figure 1 shows the locomotor activity of mice after the single intraperitoneal administration of 100 μg/kg and 1 mg/kg LPS from *Escherichia coli* (serotype 055:B5). Daytime locomotor activity was lower than that of nighttime and was not significantly changed by both 100 μg/kg and 1 mg/kg LPS administration (Fig. 1a). The administration of 1 mg/kg LPS prominently decreased nighttime locomotor activity on the first and second day and that of 100 μg/kg one was likely to reduce nighttime locomotor activity. On 6th day of the administration, nighttime locomotor activity of LPS-treated animals returned to almost control levels. The cumulative nighttime locomotor activity was significantly decreased from the first to third day of 1 mg/kg LPS administration as compared with that of the control (Fig. 1b), but the single administration of 100 μg/kg LPS did not change the cumulative nighttime locomotor activity.

**Figure 2:**
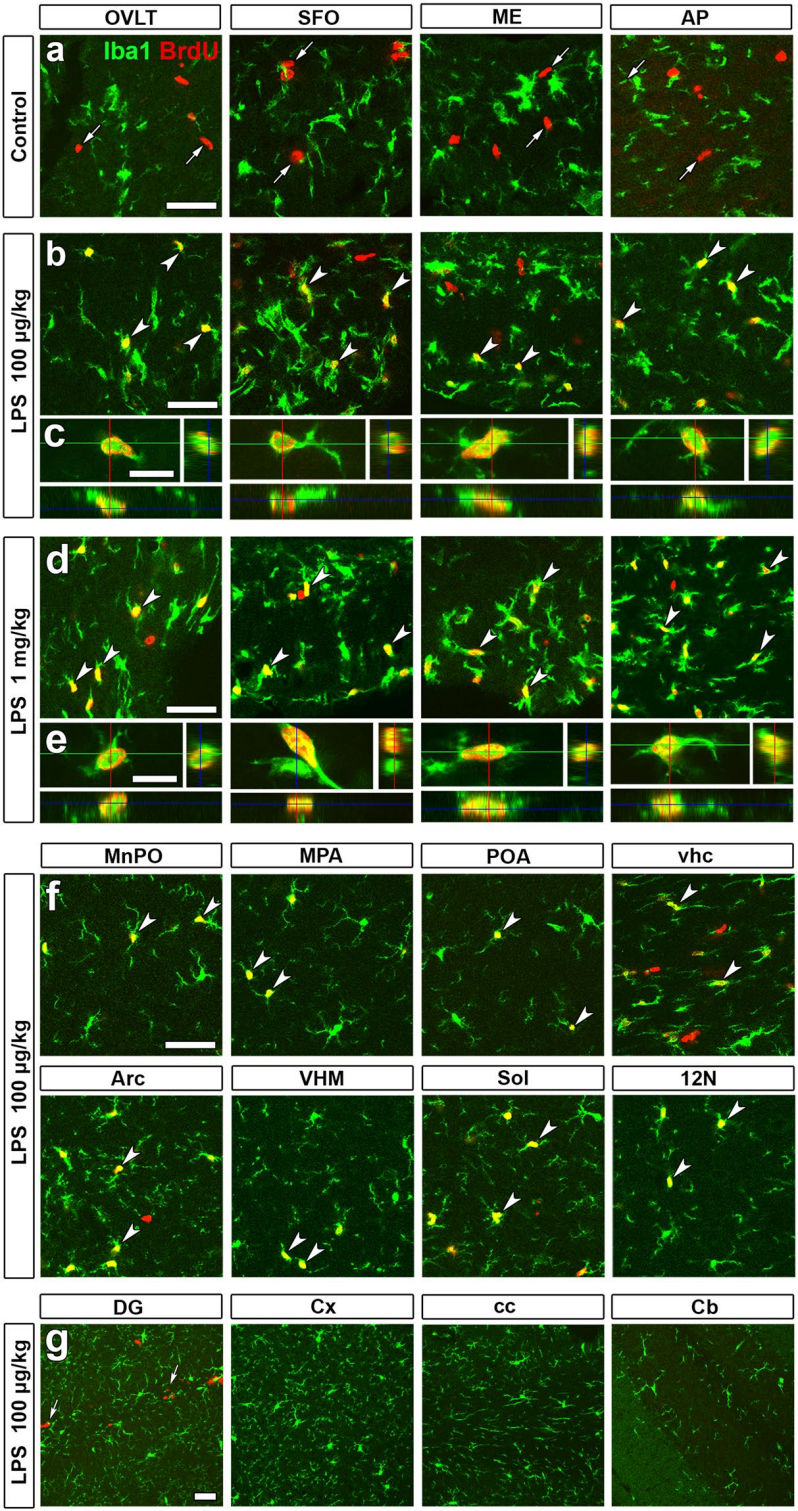
Marked increases in microglia/macrophages proliferation in the CVOs and their neighboring brain regions in the adult mouse after the single administration of LPS. Animals orally received BrdU via their drinking water (1 mg/ml) for 3 days after the single intraperitoneal administration of 100 μg/kg and 1 mg/kg LPS (serotype 055:B5). BrdU^+^ Iba1-negative cells (arrows) were neural stem/progenitor cells and endothelial cells in the CVOs of control animals (**a**). BrdU^+^ nuclei were frequently observed in Iba1^+^ microglia/macrophages in the CVOs (arrowheads) after the single intraperitoneal administration of 100 μg/kg (**b**,**c**) and 1 mg/kg (**d**,**e**) LPS. They were also seen in Iba1^+^ microglia/macrophages (arrowheads) in neighboring brain regions to the CVOs after the single intraperitoneal administration of 100 μg/kg LPS (**f**), but not in brain regions distant from the CVOs (**g**). Scale bars = 50 (**a**,**b**,**d**,**f**,**g**) and 10 (**c**,**e**) μm.

Low magnification views showed that the single intraperitoneal administration of 100 μg/kg LPS from *Escherichia coli* (serotype 055:B5) increased the number of 5-bromo-2′-deoxyuridine (BrdU)^+^ cells in the CVOs and their neighboring brain regions as compared with that of the control (Fig. S1a,b). High magnification views showed that the single intraperitoneal administration of LPS largely increased the number of BrdU^+^ and Iba1^+^ microglia/macrophage in the CVOs at a dose of 100 μg/kg (Fig. 2b) and 1 mg/kg (Fig. 2d) as compared with that of the control (Fig. 2a). A three dimensional (3D) analysis showed the presence of BrdU^+^ nuclei within Iba1^+^ microglia/macrophage in the CVOs (Fig. 2c,e). It is noted that BrdU^+^ nuclei were often seen at Iba1-negative cells in the CVOs of control mice (Fig. 2a). These BrdU^+^ Iba1-negative cells were neural stem/progenitor cells and endothelial cells, since continuous angiogenesis and neurogenesis are demonstrated in the CVOs by our previous reports^15,38^. The number of BrdU^+^ Iba1-negative neural stem/progenitor cells and endothelial cells was decreased by LPS administration as reported by our previous study^15^.

In addition to the CVOs, BrdU^+^ nuclei were frequently seen in Iba1^+^ microglia/macrophage in their neighboring brain regions at a dose of 100 μg/kg LPS: the median preoptic nucleus (MnPO), medial preoptic area (MPA), preoptic area (POA), ventral hippocampal commissure (vhc), arcuate nucleus (Arc), ventromedial hypothalamic nucleus (VMH), solitary tract (Sol), and hypoglossal nucleus (12 N) (Fig. 2f). On the other hand, BrdU^+^ and Iba1^+^ microglia/macrophages were rarely observed in the brain regions distant from the CVOs at a dose of 100 μg/kg LPS: the dentate gyrus (DG), Cx, corpus callosum (cc), and cerebellum (Cb) (Fig. 2g). The location of examined brain regions was depicted in the scheme of sagittal mouse brain section (Fig. S2). The number of BrdU^+^ and Iba1^+^ microglia/macrophage in the CVOs was also increased by the single intraperitoneal administration of LPS from *Escherichia coli* (serotype 0111:B4; Fig. S3a,b) and *Salmonella enterica* (serotype typhimurium; Fig. S3c,d).

**Figure 3:**
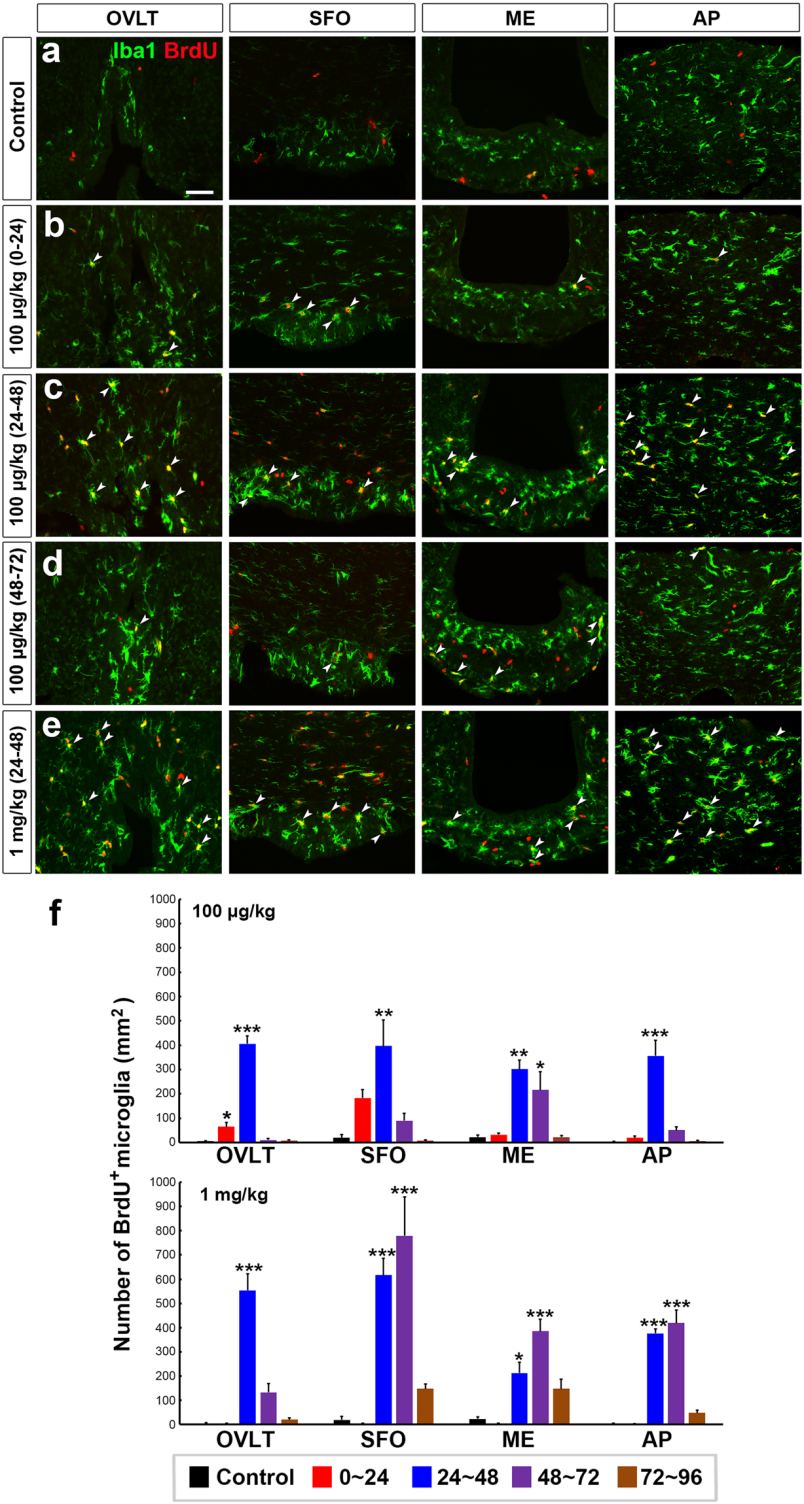
Time course changes in microglial proliferation in the CVOs of the adult mouse after the single intraperitoneal administration of LPS. Animals orally received BrdU via their drinking water (1 mg/ml) over 24 h interval after the administration of 100 μg/kg and 1 mg/kg LPS (serotype 055:B5). There was no BrdU^+^ and Iba1^+^ microglia/macrophages in the control (**a**). Many BrdU^+^ and Iba1^+^ microglia/macrophages (arrowheads) were seen in the CVOs at 24–48 h (**c**) after the administration of 100 μg/kg LPS, whereas only a few BrdU^+^ and Iba1^+^ microglia/macrophages were observed at 0–24 (**b**) and 48–72 h (**d**) except the ME. The single administration of 1 mg/kg LPS also induced robust increase in BrdU^+^ and Iba1^+^ microglia/macrophages (arrowheads) at 24–48 h (**e**). Scale bar = 50 μm. The quantitative analysis revealed that the number of BrdU^+^ and Iba1^+^ microglia/macrophages was transiently increased after the single administration of 100 μg/kg and 1 mg/kg (**f**) LPS. Data were expressed as mean (±SE) from 4 animals. *P < 0.05, **P < 0.01, ***P < 0.001 vs the control by ANOVA with Tukey post hoc test.

To determine time course of the proliferation of microglia/macrophage, time-lapse labeling of BrdU was performed. A substantial number of BrdU^+^ and Iba1^+^ microglia/macrophage were observed in the OVLT, SFO, and AP at 24–48 h (Fig. 3c), but not at 0–24 (Fig. 3b) and 48–72 h (Fig. 3d) and the control (Fig. 3a) after the single administration of 100 μg/kg LPS. In the ME, however, a considerable number of BrdU^+^ and Iba1^+^ microglia/macrophage were seen at 24–72 h (Fig. 3a–d). The number of BrdU^+^ and Iba1^+^ microglia/macrophage was also considerably increased 24–48 h after the single administration of 1 mg/kg LPS (Fig. 3e). The quantitative analysis revealed the single administration of 100 μg/kg significantly elevated the number of BrdU^+^ and Iba1^+^ microglia/macrophage in the CVOs with a peak at 24–48 h (Fig. 3f). The single administration of 1 mg/kg LPS also increased the number of BrdU^+^ and Iba1^+^ microglia/macrophage in the CVOs at 24–48 and 48–72 h (Fig. 3f). The increase of BrdU^+^ and Iba1^+^ microglia/macrophage was larger in number and lasted longer in animals administered with 1 mg/kg LPS than those with 100 μg/kg one. There were no apoptotic microglia/macrophage in the CVOs at 12, 24, and 48 h after a single administration of 1 mg/kg LPS by using TUNNEL assay (Fig. S4).

**Figure 4:**
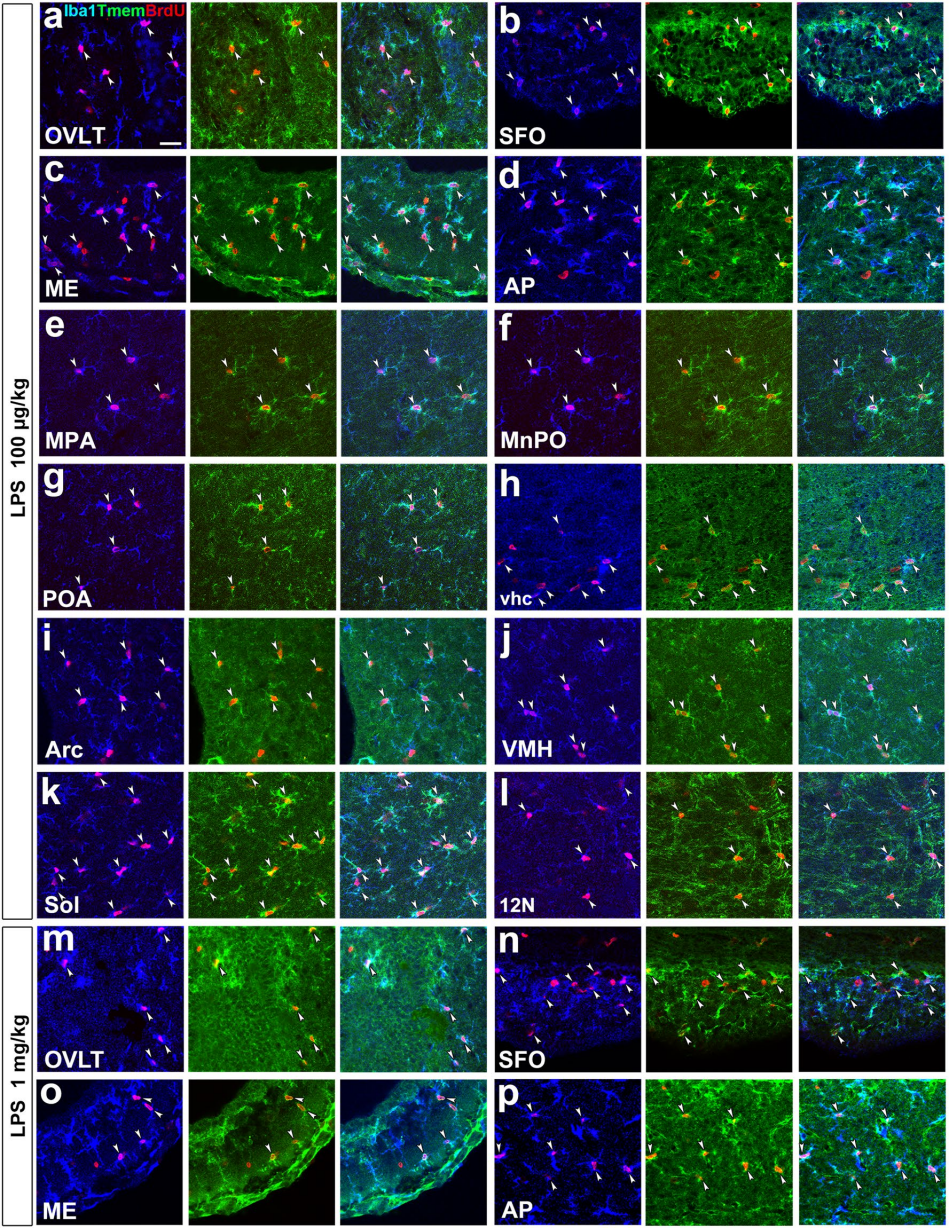
Demonstration for the proliferation of parenchyma microglia in the CVOs and their neighboring brain regions of the adult mouse after the single administration of LPS. Mice orally received BrdU via their drinking water (1 mg/ml) after the single administration of 100 μg/kg and 1 mg/kg LPS (serotype 055:B5) and fixed 3 days later for the immunohistochemistry of Tmem119. Triple labeling immunohistochemistry showed the presence of BrdU^+^ nuclei (arrowheads) in Iba1^+^ and Tmem119^+^ microglia after the single administration of 100 μg/kg (**a**–**l**) and 1 mg/kg (**m**–**p**) LPS. Scale bar = 50 μm.

To determine whether BrdU^+^ proliferating microglia/macrophages are parenchyma microglia or bone marrow-derived immune cells, we used a microglia-specific marker, trans-membrane protein 119 (Tmem119), to distinguish microglia from other immune cells^39^. The immunoreactivity of Tmem119 was observed at Iba1^+^ microglia at brain parenchyma, but not at Iba1^+^ macrophage in perivascular space (Fig. S5), indicating the Tmem119 antibody used specifically recognizes microglia. Double labeling immunohistochemistry revealed that BrdU^+^ nuclei was often observed in Tmem119^+^ and Iba1^+^ microglia in the CVOs (Fig. 4a–d) and their neighboring brain regions (Fig. 4e–l) at the dose of 100 μg/kg LPS. Likewise, BrdU^+^ and Iba1^+^ cells frequently expressed Tmem119 in the CVOs (Fig. 4m–p) and their neighboring brain regions (Fig. S6) at the dose of 1 mg/kg LPS. 3D analysis revealed the presence of BrdU^+^ nuclei within Tmem119^+^ and Iba1^+^ microglia in the CVOs and their neighboring brain regions at the dose of 100 μg/kg **(**Fig. S7) and 1 mg/kg (Fig. S8) LPS.

The results of the quantitative analysis showed that BrdU^+^ and Iba1^+^ cells almost exclusively expressed Tmem119 in the CVOs (percentage of Tmem119 expression in BrdU^+^ and Iba1^+^ cells: OVLT, 97.00 ± 1.53; SFO, 97.10 ± 2.90; ME, 97.96 ± 1.03; AP, 95.38 ± 1.54) and their neighboring brain regions (MPA, 98.77 ± 1.23; MnPO, 95.58 ± 2.30; POA, 98.48 ± 1.52; vhc, 91.47 ± 2.24; Arc, 98.74 ± 0.64; VMH, 99.02 ± 0.98; Sol, 99.23 ± 0.72; 12 N, 96.08 ± 3.92) at dose of 100 μg/kg LPS. Similarly, almost all of BrdU^+^ and Iba1^+^ cells in the CVOs were positive for Tmem119 (percentage of Tmem119 expression in BrdU^+^ and Iba1^+^ cells: OVLT, 95.62 ± 0.56; SFO, 93.55 ± 2.15; ME, 99.33 ± 0.67; AP, 97.69 ± 0.60) and their neighboring brain regions (MPA, 98.15 ± 1.85; MnPO, 96.49 ± 3.51; POA, 97.92 ± 2.08; vhc, 98.10 ± 1.90; Arc, 98.33 ± 1.67; VMH, 99.12 ± 0.88; Sol, 98.53 ± 0.74; 12N, 95.33 ± 1.17) when animals received the single intraperitoneal administration of 1 mg/kg LPS. These results indicate that BrdU^+^ proliferating Iba1^+^ cells in the brain are definitely microglia during LPS-induced inflammation.

**Figure 5:**
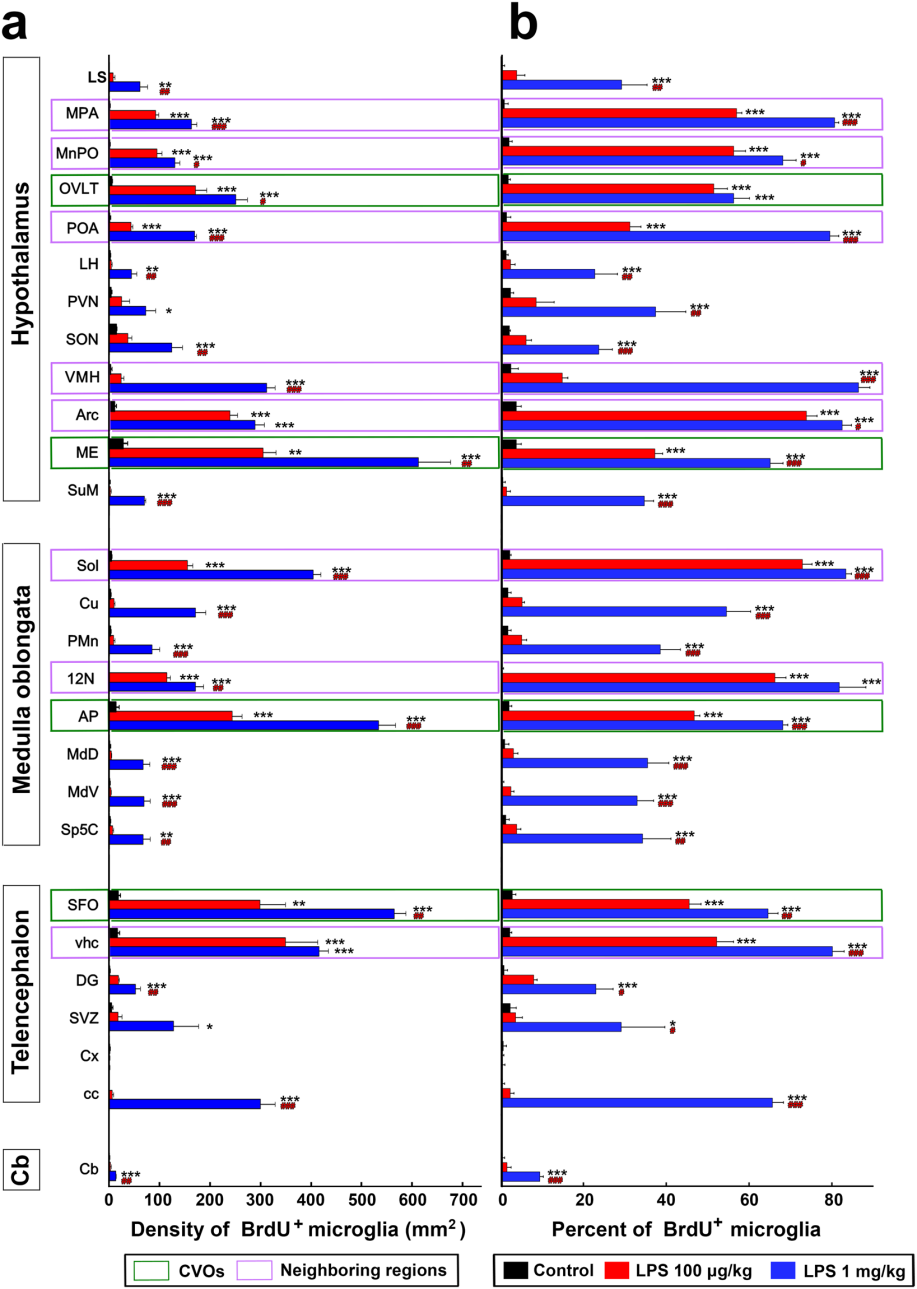
A quantitative analysis revealing heterogeneous microglial proliferation in the adult mouse brains after the single administration of 100 μg/kg and 1 mg/kg LPS (serotype 055:B5). The density (**a**) and percentage (**b**) of BrdU^+^ Iba1^+^ microglia were significantly increased in the CVOs and their neighboring brain regions, but not in brain regions distant from the CVOs, after the single administration of 100 μg/kg LPS. In the case of 1 mg/kg LPS, the density and percentage of BrdU^+^ and Iba1^+^ microglia were increased in the brain region being distant from the CVOs, in addition to the CVOs and their neighboring regions. Data were expressed as the mean (±SE) of 4 animals. *P < 0.05, **P < 0.01, ***P < 0.001 vs the control and ^#^P < 0.05, ^##^P < 0.01, ^###^P < 0.001 between 100 μg/kg and 1 mg/kg LPS by an ANOVA with Tukey post hoc test.

The results of the quantitative analysis showed that the single administration of 100 μg/kg LPS significantly (P < 0.05) increased the density of BrdU^+^ microglia in the CVOs: the OVLT (number of BrdU^+^ and Iba1^+^ microglia/mm^2^, analyzed optical thickness of 4 µm; control, 5.53 ± 1.06; LPS, 171.35 ± 22.00), ME (control, 28.31 ± 8.34; LPS, 305.09 ± 25.34), AP (control, 14.30 ± 5.48; LPS, 243.52 ± 19.45), and SFO (control, 18.14 ± 4.95; LPS, 299.13 ± 50.00) (Fig. 5a). The single administration of 100 μg/kg LPS significantly (P < 0.05) increased the density of BrdU^+^ microglia in the brain regions neighboring the CVOs such as the MPA, MnPO, POA, VMH, Arc, Sol, 12 N, and vhc (Fig. 5a). On the other hand, the single administration of 100 μg/kg LPS did not significantly (P > 0.05) increase the density of BrdU^+^ microglia in brain regions distant from the CVOs, such as the lateral septal nucleus (LS), lateral hypothalamic area (LH), paraventricular nucleus (PVN), supraoptic nucleus (SON), supramammillary nucleus (SuM), cuneate nucleus (Cu), paramedian reticular nucleus (PMn), dorsal part of the medullary reticular nucleus (MdD), ventral part of the medullary reticular nucleus (MdV), caudal part of the spinal trigeminal nucleus (Sp5C), DG, subventricular zone (SVZ), Cx, cc, and Cb (Fig. 5a). Upon the single administration of 100 μg/kg LPS, the percentage of Iba1^+^ microglia that are also positive for BrdU was significantly (P < 0.001) increased in the CVOs and their neighboring brain regions (Fig. 5b).

The single administration of 1 mg/kg LPS significantly (P < 0.05) increased the density of BrdU^+^ microglia in the CVOs and their neighboring brain regions, as was also the case for 100 μg/kg LPS (Fig. 5). In contrast to 100 μg/kg LPS, the administration of 1 mg/kg LPS significantly (P < 0.05) increased the density of BrdU^+^ microglia in brain regions distant from the CVOs: the LS (control, 0.48 ± 0.48; LPS 61.29 ± 14.79), LH (control, 2.22 ± 0.74; LPS 44.31 ± 11.18), PVN (control, 4.43 ± 1.48; LPS 73.11 ± 19.23), SON (control, 14.62 ± 1.16; LPS 124.49 ± 21.14), SuM (control, 0.98 ± 0.98; LPS 69.91 ± 2.61), Cu (control, 2.95 ± 1.21; LPS 170.87 ± 20.60), PMn (control, 2.95 ± 1.21; LPS 85.66 ± 15.01), MdD (control, 1.48 ± 1.48; LPS 67.94 ± 12.93), MdV (control, 1.35 ± 0.79; LPS 69.42 ± 12.09), Sp5C (control, 2.22 ± 1.41; LPS 67.20 ± 14.93), DG (control, 1.23 ± 1.23; LPS 53.43 ± 9.68), SVZ (control, 4.86 ± 2.82; LPS 127.84 ± 49.72), cc (control, 0.40 ± 0.40; LPS 299.95 ± 29.15), and Cb (control, 0.74 ± 0.74; LPS 13.29 ± 1.48) (Fig. 5a). The percentage of Iba1^+^ microglia that are also positive for BrdU was also higher in many brain regions after the administration of 1 mg/kg LPS than the control (Fig. 5b). It is surprising that the single administration of both 100 μg/kg and 1 mg/kg LPS did not significantly increase the density and percentage of BrdU^+^ and Iba1^+^ microglia in the Cx as compared with the control.

**Figure 6:**
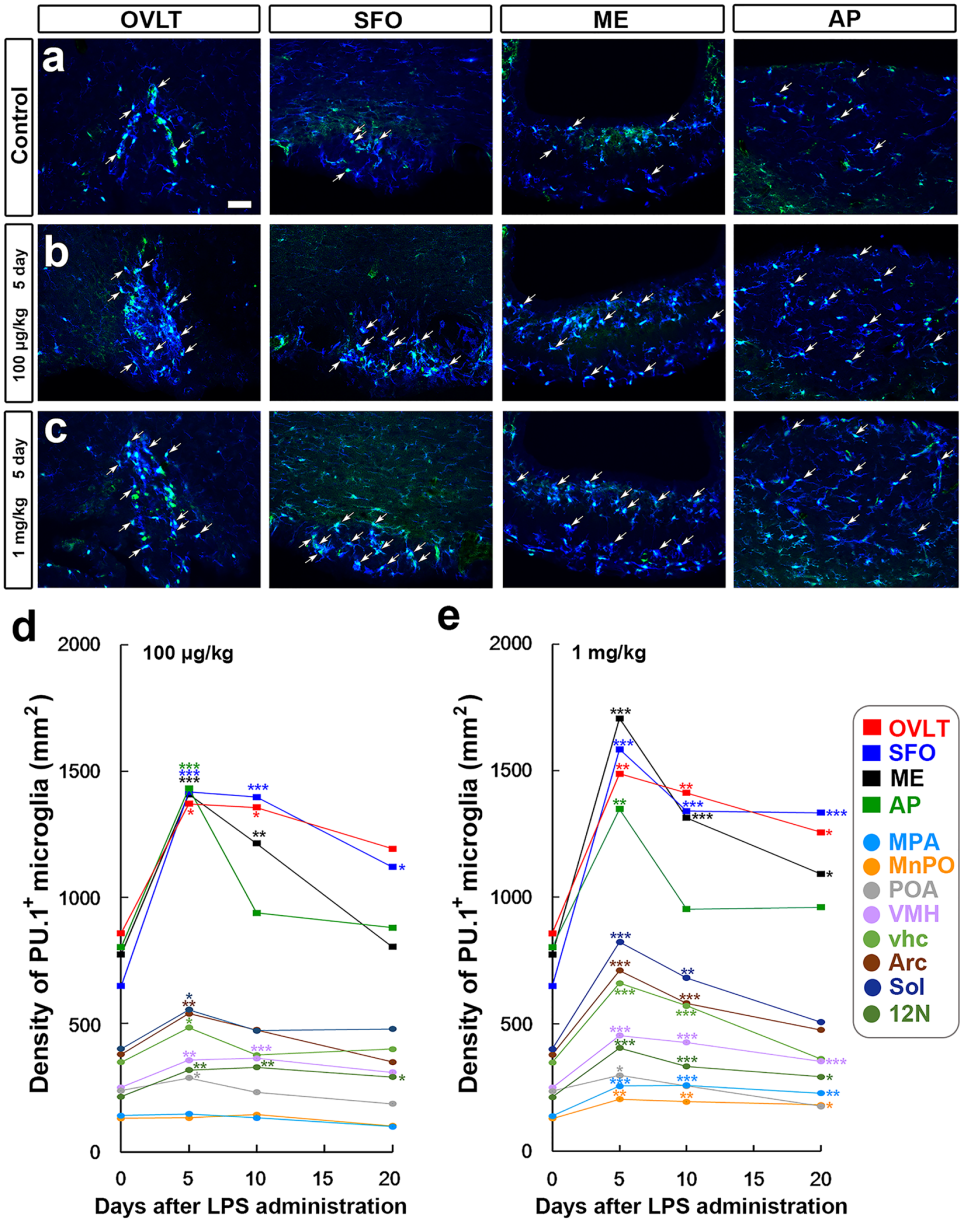
Changes in microglial density in CVOs in the adult mouse brain after the single administration of 100 μg/kg and 1 mg/kg LPS (serotype 055:B5). The density of PU.1^+^ and Iba1^+^ microglia (arrowheads) was higher in the CVOs on the 5th day after the single administration of 100 μg/kg (**b**) and 1 mg/kg (**c**) LPS than that of the control (**a**). Scale bar = 50 μm. A quantitative analysis showing changes in PU.1^+^ and Iba1^+^ microglia density in CVOs and their neighboring brain regions in the adult mouse brain after the single administration of 100 μg/kg (**d**) and 1 mg/kg (**e**) LPS (serotype 055:B5). The density of PU.1^+^ and Iba1^+^ microglia was significantly increased in the CVOs and neighboring brain regions to the CVOs on the 5th and 10th day after the single administration of 100 μg/kg or 1 mg/kg LPS. Microglial density decreased on the 20th day after the LPS administration. Data were expressed as the mean (±SE) of 4 animals. *P < 0.05, **P < 0.01, ***P < 0.001 vs the control by an ANOVA with Tukey post hoc test.

In order to clarify whether the LPS-induced proliferation of microglia actually result in increased density of them, we examined time-dependent changes in microglial density using microglial nuclear marker PU.1 and cytoplasmic marker Iba1. The density of PU-1^+^ and Iba1^+^ microglia was higher in the CVOs on the 5th day after the single administration of 100 μg/kg (Fig. 6b) and 1 mg/kg (Fig. 6c) LPS than that in the control (Fig. 6a). The quantitative analysis revealed that the density of PU.1^+^ and Iba1^+^ microglia was greater in the CVOs than in other brain regions in control mice following the administration of 100 μg/kg LPS (Fig. 6d). The single administration of 100 μg/kg LPS significantly (P < 0.05) increased the density of PU-1^+^ and Iba1^+^ microglia in the CVOs: the OVLT (number of PU.1^+^ and Iba1^+^ microglia/mm^2^; analyzed thickness of optical slices, 4 μm: control, 857.44 ± 77.88; 5 day, 1371.06 ± 123.09; 10 day, 1356.20 ± 140.37), SFO (control, 648.16 ± 21.81; 5th day, 1416.94 ± 72.86; 10^th^ day, 1397.11 ± 146.41), ME (control, 773.53 ± 26.58; 5th day, 1408.56 ± 68.06; 10th day, 1213.74 ± 76.06), and AP (control, 802.13 ± 26.64; 5th day, 1431.91 ± 63.12; 10th day, 938.07 ± 46.93). The single administration of 100 μg/kg LPS also significantly (P < 0.05) increased the density of PU.1^+^ and Iba1^+^ microglia in the brain regions neighboring the CVOs: the MPA, POA, VMH, Arc, Sol, 12 N, and vhc. The density of PU.1^+^ and Iba1^+^ microglia returned to control levels on the 20th day, but did not in the SFO and 12 N.

The single administration of 1 mg/kg LPS significantly (P < 0.05) increased the density of PU.1^+^ and Iba1^+^ microglia in the CVOs and their neighboring brain regions (Fig. 6e). The pattern of time-dependent changes in Iba1^+^ microglial density were similar to those following the administration of 100 μg/kg LPS. However, the increase in microglia density was relatively larger in the animals administered with 1 mg/kg LPS than that with 100 μg/kg LPS. The microglia density in the ME, AP, Arc, MnPO, and MPA on the 20th day after the single administration of 1 mg/kg LPS were still significantly higher than those in the control.

**Figure 7:**
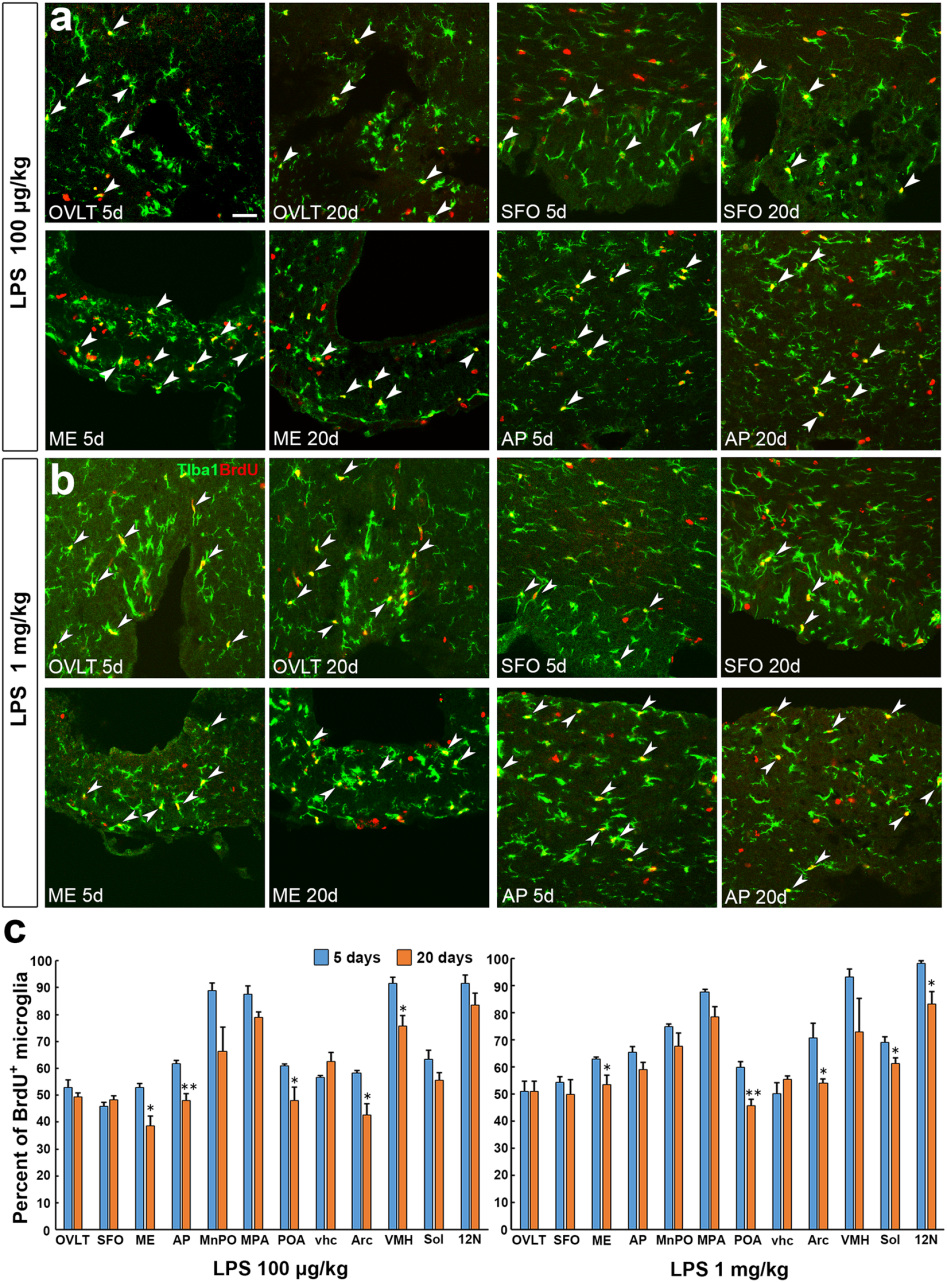
Survival of BrdU-labeled microglia in the CVOs and their neighboring brain regions in the adult mouse brain on 20th day after the single administration of LPS. Animals orally received BrdU via their drinking water (1 mg/ml) for 5 days after the administration of 100 μg/kg and 1 mg/kg LPS (serotype 055:B5) and fixed on 5th and 20th day after the LPS stimulation. Representative confocal images showed that many BrdU^+^ and Iba1^+^ microglia were seen on 20th day after the LPS stimulation as on 5th day (**a**,**b**). Scale bar = 50 μm. The percentage of BrdU-labeled microglia was significantly lower in the POA, ME, Arc, and AP on 20th day after 100 μg/kg LPS than that on 5th day (**c**). The percentage of BrdU-labeled microglia was significantly lower in the POA, ME, Arc, and on 20th day after 1 mg/kg LPS than that on 5th day (**b**). Data were expressed as the mean (±SE) of 5 animals. *P < 0.05, **P < 0.01 vs the control by an unpaired Student’s t-test.

To determine whether decrease of microglia density is due to loss of proliferated or non-proliferated microglia, we examined the percentage of BrdU^+^ and Iba1^+^ microglia in total microglia on 5th and 20th day after the single administration of LPS. BrdU^+^ and Iba1^+^ microglia were still abundantly seen in the CVOs (Fig. 7a,b) and their neighboring brain regions (Fig. S9, S10) on the 20th day after the single administration of 100 μg/kg and 1 mg/kg LPS. The quantitative analysis revealed that the percentage of BrdU^+^ and Iba1^+^ microglia in total microglia was slightly lower in the POA, ME, Arc, and AP on the 20th day as compared with the 5th day after the single administration of 100 μg/kg LPS (Fig. 7c). Similarly, the percentage in BrdU-labeling of microglia was marginally lower in the POA, ME, Arc, and AP on the 20th day as compared with the 5th day following the single administration of 1 mg/kg LPS (Fig. 7c).

## Discussion

The results obtained herein provide evidence for marked increases in microglial proliferation in the CVOs and their neighboring brain regions during mild inflammation induced by a single systemic LPS stimulation at a dose of 100 μg/kg. Moreover, microglial proliferation was found to be induced in many other brain regions as like in these regions during severe inflammation caused by a systemic single LPS stimulation at a dose of 1 mg/kg. LPS-induced increase of resident microglia proliferation leaded to a transient expansion of the microglial population in the brain. These results indicate that transient and marked increases of the microglial population generally occur in many brain regions during infection-induced inflammation and propose a new aspect for microglial functions in brain inflammation and pathogenesis of brain diseases.

In the present study, we used Tmem119 to distinguish microglia from perivascular macrophages and infiltrating leukocytes. Tmem119 is shown to label only resident microglia, allowing for a clear distinction between resident and infiltrating leukocytes after LPS-induced inflammation and traumatic injury in the optic nerve with monocyte influx^39^. Engrafted bone marrow myeloid cells does not express Tmem119 even 6 months after they infiltrate the brain and transform into microglia^39^. In demyelinating lesions of MS patients, it is shown that Tmem119 is expressed on microglia but not on infiltrating leukocytes and average 45% of the macrophage-like cells in active lesions are derived from resident microglia pool^40,41^. A subpopulation of leukocytes firstly appears at fluid-filled compartments such as the velum interpositum and ambient and basal cisterns, and then they infiltrate to reside at perivascular and periventricular spaces at early phase of EAE^42,43^. The present Tmem119 immunohistochemistry showed that BrdU^+^ proliferating microglia often expressed a microglia-specific marker Tmem119 upon peripheral LPS stimulation. Moreover, the present study revealed that BrdU^+^ and Tmem119^+^ microglia were evenly distributed at brain parenchyma in the CVOs rather than localized specifically at perivascular and/or periventricular spaces. Therefore, it is probable that proliferating microglia in the CVOs and their neighboring regions essentially arise from resident microglia pool, although we cannot deny the possibility that a subpopulation of perivascular macrophage or infiltrating leukocytes is also divided, since there are a few Tmem119-negative Iba1^+^ cells.

A whole brain analysis reveals that microglial proliferation occurs at a rate of 10.1% per week with the intraperitoneal administration of 1 mg/kg LPS every other day^7^. Whole brain analysis has technical limitation to elucidate brain region-dependent heterogeneity of microglia proliferation. Our previous study has revealed that the single stimulation of high dose (1 mg/kg) of LPS induced marked increases in microglial proliferation in the CVOs^15^. The present study newly demonstrated microglia-proliferating brain regions by the single LPS stimulation of a low dose of 100 μg/kg: the brain regions neighboring the CVOs such as the MPA, MnPO, POA, vhc, Arc, VMH, Sol, and 12N. These brain regions are known to play roles in thermoregulatory and autonomic functions^44,45^. Moreover, the present study showed that the single LPS stimulation at a high dose of 1 mg/kg induced microglial proliferation in many other brain regions as in the CVOs and their neighboring regions: such as the LS, LH, PVN, SON, SuM, Cu, PMn, MdD, MdV, Sp5C, DG, SVZ, cc, and Cb. On the other hand, proliferation of microglia was not observed in the Cx after the single administration of LPS. This result is well coincided with the previous report to demonstrate the absence of microglial proliferation in Cx even after the consecutive four-day administration of 1 mg/kg LPS^14^. Moreover, microglia proliferation in the DG and Cb was absent and a few after the single administration of 100 μg and 1 mg/kg LPS, respectively. Microglia in the Cx possess a proliferative capability, since microglial in the Cx are shown to proliferate in response to more severe inflammatory stimulation, ischemic injury^46^. In the Cx, resident microglia, but not infiltrating bone marrow-derived immune cells, are identified as the first cell population to proliferate after traumatic injury^46^. Although circulating LPS are not able to pass impermeable BBB, recently it is shown that LPS is incorporated mainly in ependymal and endothelial cells in the CVOs through lipoprotein transport system^24^. Expression of TLR4 is reported to be markedly higher in the CVOs than other brain regions^25–27^. Moreover, the activation of proinflammatory TLR4-associated signaling molecules, STAT3 and NF-κB, occurs in the CVOs after the single systemic administration of LPS^27–29^. The *in situ* hybridization histochemistry shows that mRNA expression of proinflammatory cytokines, IL-1 and IL-6, is increased preferentially in the CVOs after a single systemic administration of LPS^47,48^. Therefore, the CVOs are the blood-brain interface to initiate early stage of brain inflammation probably via direct interaction between LPS and TLR4. Taken together, our results demonstrate that increase in microglia proliferation during LPS-induced inflammation non-uniformly occurs region- and dose-dependent manner, indicating diversity of microglia sensitivity for proliferation. Moreover, it is evident that increase of microglia proliferation is caused by the LPS administration of a low dose of 100 μg/kg, which suggests dynamic changes of microglia population under mild inflammation such as common cold.

The present study showed that the density of microglia in the CVOs increased by 81% in the OVLT, 75% in the ME, 67% in the ME, and 36% in the SFO on the 5th day after the single stimulation of low dose (100 μg/kg) of LPS. Similarly, the density of microglia in their neighboring brain regions was increased by 79% in the MnPO, 56% in the MPA, 37% in the POA, 81% in the vhc, 76% in the Arc, 92% in the VMH, 72% in the Sol, and 148% in the 12N. An increase in microglial density was also observed following the single stimulation of high dose (1 mg/kg) of LPS. Moreover, cumulative number of BrdU^+^ microglia in the CVOs was apparently larger in the mice receiving time-lapse BrdU-labeling for 3 days (Fig. 3) than the animals that receiving continuous 3-day BrdU drinking (Fig. 5). This observation suggests that daughter microglial cells arise from a subpopulation of microglia after a single administration of LPS. The density of brain microglia returns to normal levels within 1 week of inhibitor cessation through proliferation of potential microglial progenitor cells after elimination of ∼99% of microglia by CSF1R antagonist^49^. Microglial density almost returned to control levels on the 20th day after the single stimulation of 100 μg/kg and 1 mg/kg LPS and BrdU^+^ proliferated microglia survived as similar rate as non-proliferated ones. This observation suggests that microglia probably undergo apoptosis to return their density to normal control levels. In the mouse and human brain, the microglial density remains remarkably stable over a lifetime, which is maintained by coupled proliferation and apoptosis of resident microglia^9^. Thus, the present study demonstrates for the first time that a transient increase in the microglial population of the brain occurs during LPS-induced inflammation.

The present study showed that LPS-induced microglial proliferation was limited in the CVOs and their neighboring brain regions by the LPS administration at a low dose of 100 μg/kg, whereas the proliferation of microglia was also observed in other many brain regions by the administration of high dose (1 mg/kg) of LPS. The dose-dependent difference of microglia proliferation is associated with that of locomotor activity, an indicator of sickness behavior. The stimulation of low dose (100 μg/kg) of LPS marginally reduced the locomotor activity only on the first day, while reduced locomotor activity by high dose (1 mg/kg) of LPS stimulation was robust and long-lasting. The intraperitoneal administration of low dose (100 μg/kg) of LPS is shown to induce hyperthermia in mice, while that of high dose (1 mg/kg) of LPS causes hypothermia^37^. Thus, dose-dependent difference of microglia proliferation is probably due to that of neural circuits controlling LPS-induced sickness behaviors and thermoregulation.

It is known that microglial diversity enables region-specific sensitivities to environmental cues to sustain regionally localized homeostatic functions^50–52^. The CVOs, a specialized group of brain regions, lack a normal BBB and, thus, play a pivotal role in blood-brain communication^21^. Cytokines and pathogens reach the CVOs by the fenestrated capillaries of the CVOs and initiate inflammatory responses of the brain via STAT3 and NF-κB signaling in the CVOs^27–29,45^. The mRNA signals of IkBa are first detected in parenchyma cells in the CVOs and then gradually spread to vascular and parenchyma cells throughout the entire brain^29^. The brain regions neighboring the CVOs such as the MPA, MnPO, POA, VMH, Arc, Sol, and 12 N have been reported to participate in LPS-induced inflammatory and thermoregulatory responses and behaviors^44,45^. Thus, inflammatory responses such as the activation of STAT3 and NF-κB signaling and the production of cytokines are more marked in the CVOs and their neighboring brain regions than other brain regions^27–29,45,47,48^. Activated microglia are able to mediate the clearance of pathogens, cytokines, and toxic factors^48^. Fractalkine receptor-deficient mice show a prolonged duration of depression-like behaviors during LPS-induced inflammation by a persistent activated microglial phenotype in the hippocampus and prefrontal cortex^53^. In Alzheimer’s disease, β-amyloid plaques are tightly enveloped by microglia processes and microglia constitute a barrier that prevents outward plaque expansion^54^. The present study revealed the absence of microglia apoptosis in the CVOs. Therefore, increase in microglial population is possibly related to the maintenance of brain homeostasis from LPS-induced inflammatory disturbances rather than replacement of apoptotic microglia.

## Materials and Methods

### Animals and Treatments

Adult male mice (C57BL/6J) aged 70–84 days old were used in the present experiments. They were housed in a colony room with a 12-h light/dark cycle; light on at 7:00 and light off at 19:00. Animals were given *ad libitum* access to commercial chow and tap water. As the inflammatory stimulation, mice received a single intraperitoneal administration of LPS from *Escherichia coli* (serotype 055:B5 and 0111:B4; Sigma-Aldrich) and *Salmonella enterica* (serotype typhimurium; Sigma-Aldrich) at a dose of either 100 μg/kg or 1 mg/kg in pyrogen-free physiological saline (Otsuka Pharmaceutical Co. LTD., Tokushima, Japan). Regarding BrdU labeling, mice received BrdU (1 mg/ml) orally for 3 days through their drinking water to examine brain region-dependent heterogeneity and dose-dependent difference of LPS-induced microglia proliferation. Animals also received BrdU drinking (1 mg/ml) for 5 days after the administration of LPS to investigate survival of divided microglia. For time-lapse labeling of BrdU, mice received BrdU drinking (1 mg/ml) for 0–24, 24–48, 49–72, and 72–96 hr after the administration of LPS. Animal care and experiments were performed in accordance with the Guidelines laid down by the NIH and the Guideline for Proper Conduct of Animal Experiments Science Council of Japan. The experimental protocol was approved by the Animal Ethics Experimental Committee of the Kyoto Institute of Technology.

### Telemetric recording of locomotor activity

Mice were anesthetized with isoflurane and then implanted intraperitoneally with E-Mitter mouse telemetry system implants to monitor gross locomotor activity by E-Mitter mouse telemetry system (Starr Lifescience Corp., Oakmont, PA)^28^. They were then housed in a room in which the ambient temperature was set to 25 °C on a 12 h light/dark cycle. Seven days after the implantation, the animals received an intraperitoneal administration of LPS or saline, as above. LPS/saline administration was done at approximately 11:30. Change in locomotor activity was detected as change in the position of E-Mitter implants over ER4000 Energizer/Receiver board and a change in the signal strength was converted to activity pulses and analyzed by VitalView software.

### Light Microscopic Histochemistry

After deep anesthesia with isoflurane, mice were perfused transcardially with phosphate-buffered saline (PBS; pH 7.2) containing 0.1% trisodium citrate dihydrate followed by 4% paraformaldehyde (PFA) in 0.1 M phosphate buffer (PB, pH 7.4). After perfusion of the fixative, brains were dissected out, postfixed in 4% PFA in 0.1 M PB (pH 7.2) at 4 °C for 24 h, cryoprotected by 30% sucrose in PBS, and frozen quickly in Tissue-Tek OCT compound (Sakura Finetechnical, Tokyo, Japan). Sections were obtained by coronal cutting on a cryostat (Leica, Wetzlar, Germany) at a thickness of 30 μm. In single and double labeling immunohistochemistry, a standard immunofluorescence technique was performed on free-floating sections according to our previous study^15,55^. In brief, sections were washed with PBS and treated with 25 mM glycine in PBS for 20 min in order to quench the remaining aldehyde residues. The sections were then pretreated with 5% normal goat serum (NGS) in PBS containing 0.3% Triton X-100 (PBST) at 4 °C for 72 h in order to reduce the non-specific binding of IgG, and were subsequently incubated with the primary antibody at 4 °C for 48 h: rabbit IgG against ionized calcium binding adaptor molecule 1 (Iba1: macrophage/microglial cytoplasmic marker; Wako Chemicals, Osaka, Japan; dilution 1:800); guinea pig IgG against Tmem119 (microglial marker: the antigen was a synthetic peptide corresponding to mouse Tmem119 aa119–167; TIF-H28; dilution 1:400) and laminin (the antigen was laminin-111 from Engellbreth-Holm-Swarm murine sarcoma basement membrane; YI-2008; dilution 1:200)^56^; mouse IgG against PU.1 (microglial nuclear marker: Spi-1, Santa Cruz Biotechnology, Santa Cruz, CA; dilution 1:20).

For detection of apoptosis cells, we employed TUNNEL method by using Apoptosis *in situ* Detection Kit (293–71501, Wako Chemical, Osaka, Japan). For Tmem119, the cryosections were treated with 0.05% citraconic anhydride solution (Immunosaver; Nisshin EM Co., Tokyo, Japan) for 15 min at 95 °C before the incubation of the primary antibody against Tmem119. This was followed by a treatment with Alexa488- or Alexa594-conjugated goat IgG (Jackson ImmunoResearch Laboratories, West Grove, PA; dilution 1:400) against rabbit or goat IgG in PBST for 2 h. In the case of mouse primary antibody, however, the sections were pretreated with unlabeled goat Fab fragment against mouse IgG (Jackson ImmunoResearch; dilution 1:400) for 2 h to mask endogenous mouse IgG-like proteins and Alexa488-conjugated goat F(ab)^2^ against mouse IgG was used (Jackson ImmunoResearch; dilution 1:100) to avoid nonspecific binding of endogenous mouse Fc receptors. The sections were treated with 2 N HCl at 37 °C for 20 min followed by 0.1 M borate buffer (pH 8.4) for 20 min, and then incubated at 4 °C for 24 h. The sections were incubated with rat IgG against BrdU (Abcam, Cambridge, UK, AB6326; dilution 1:1,000) at 4 °C for 48 h and then with Alexa488- or Alexa594-conjugated goat IgG (Jackson ImmunoResearch Laboratories; dilution 1:400) against rat IgG in PBST for 2 h. Regarding nuclear staining, sections were incubated with propidium iodide (40 μg/ml; Sigma-Aldrich Japan). After a brief wash with PBST, the coverslips were sealed with Vectashield (Vector Labs, Burlingame, CA) and observations were performed using laser-scanning confocal microscopes (Fluoview, FV10i, OLYMPUS, Tokyo, Japan or LSM 510, Carl Zeiss, Oberkochen, Germany). Images (2,048 × 2,048 pixels) were saved as TIF files by employing Olympus FV10-ASW Ver 1.7 Viewer or LSM 510 META Image Browser 4.2.0.121 for Windows and arranged using Photoshop CC (Adobe Systems Incorporated, San Jose, CA).

### Quantitative and Statistical Analyses

We analyzed five sections per animal from the OVLT and eight sections per animal from the other brain regions according to the mouse brain atlas^57^. The abbreviation of brain region names and location of brain regions are shown in Table and Fig. S2, respectively. 1In order to perform the quantitative analysis, confocal images were obtained under the same pinhole size (optical slice thickness of 4 µm), brightness, and contrast setting. We saved images (1,024 × 1,024 pixels) as TIF files by employing LSM 510 Image Browser 4.2.0.121 for Windows, and arranged them using Photoshop CC. The number of BrdU^+^ nuclei in Iba1^+^ microglia and the area of Iba1^+^ microglia were assessed using WinRoof, the threshold intensity of which was set to include measurement profiles by visual inspections and was kept constant. Data were expressed as the mean ± SEM. The significance of differences was assessed using a significance level of P < 0.05 by ANOVA with Tukey’s test.

### Declaration of interest

The authors declare that there is no conflict of interest that may be perceived as prejudicing the impartiality of the research reported.

### Electronic supplementary material

Supplementary Information
