# Author Correction: Brain Region-dependent Heterogeneity and Dose-dependent Difference in Transient Microglia Population Increase during Lipopolysaccharide-induced Inflammation

**DOI:** 10.1038/s41598-023-47503-z

**Published:** 2023-11-30

**Authors:** Eriko Furube, Shintaro Kawai, Haruna Inagaki, Shohei Takagi, Seiji Miyata

**Affiliations:** 1 Department of Applied Biology, Kyoto, 606-8585 Japan; 2 The Center for Advanced Insect Research Promotion, Kyoto Institute of Technology, Matsugasaki, Sakyo-ku, Kyoto, 606-8585 Japan

Correction to: *Scientific Reports* 10.1038/s41598-018-20643-3, published online 02 February 2018

The original version of this Article contained errors in Figure 2 and 4. In Figure 2, right end of panel d, where the confocal images were inadvertently derived from different confocal databases.

also, In Figure 4 there was an error in panel f, where the confocal images were duplicated from the panel e.

The correct Figure 2 and Figure 4 and their accompanying legends appear below.

**Figure 2:**
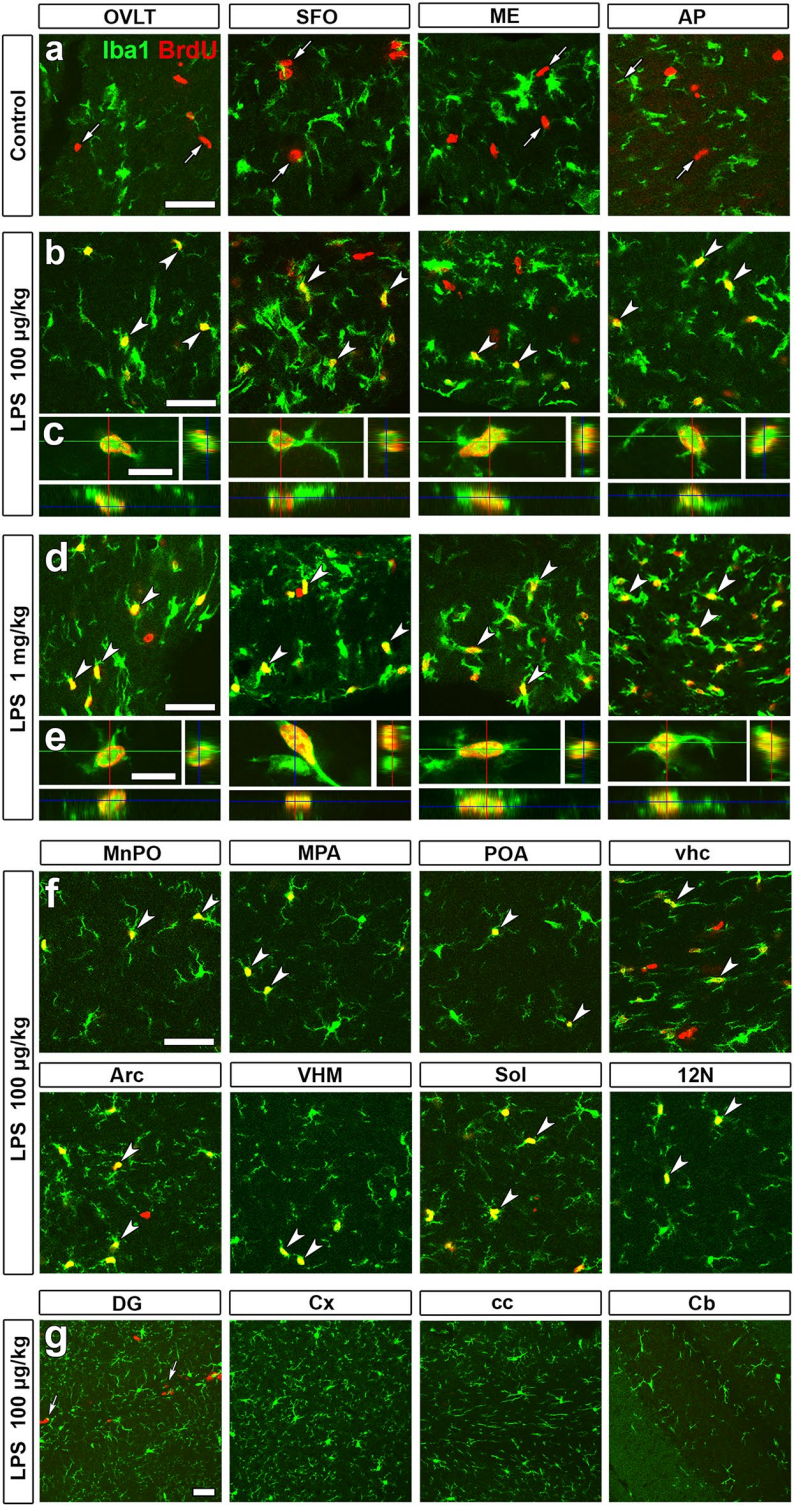
Marked increases in microglia/macrophages proliferation in the CVOs and their neighboring brain regions in the adult mouse after the single administration of LPS. Animals orally received BrdU via their drinking water (1 mg/ml) for 3 days after the single intraperitoneal administration of 100 μg/kg and 1 mg/kg LPS (serotype 055:B5). BrdU+ Iba1-negative cells (arrows) were neural stem/progenitor cells and endothelial cells in the CVOs of control animals (**a**). BrdU+ nuclei were frequently observed in Iba1+ microglia/macrophages in the CVOs (arrowheads) after the single intraperitoneal administration of 100 μg/kg (**b**,**c**) and 1 mg/kg (**d**,**e**) LPS. They were also seen in Iba1+ microglia/macrophages (arrowheads) in neighboring brain regions to the CVOs after the single intraperitoneal administration of 100 μg/kg LPS (**f**), but not in brain regions distant from the CVOs (**g**). Scale bars = 50 (**a**,**b**,**d**,**f**,**g**) and 10 (**c**,**e**) μm.

**Figure 4:**
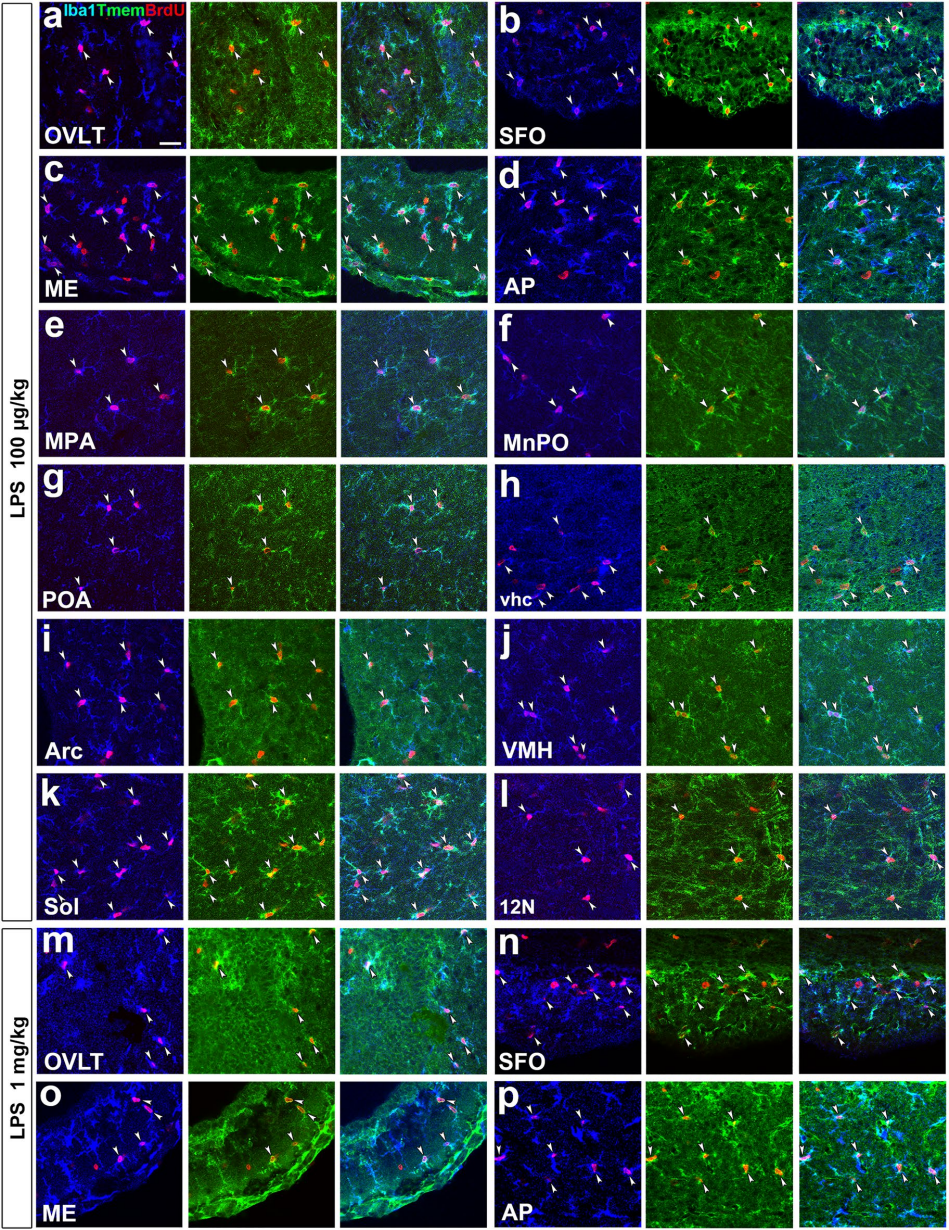
Demonstration for the proliferation of parenchyma microglia in the CVOs and their neighboring brain regions of the adult mouse after the single administration of LPS. Mice orally received BrdU via their drinking water (1 mg/ml) after the single administration of 100 μg/kg and 1 mg/kg LPS (serotype 055:B5) and fixed 3 days later for the immunohistochemistry of Tmem119. Triple labeling immunohistochemistry showed the presence of BrdU+ nuclei (arrowheads) in Iba1+ and Tmem119+ microglia after the single administration of 100 μg/kg (**a**–**l**) and 1 mg/kg (**m**–**p**) LPS. Scale bar = 50 μm.

