# Supplementary material for: Brain Region-dependent Heterogeneity and Dose-dependent Difference in Transient Microglia Population Increase during Lipopolysaccharide-induced Inflammation
Source: Sci Rep. 2018 Feb 2;8:2203. doi: 10.1038/s41598-018-20643-3 (PMC5797160; doi:10.1038/s41598-018-20643-3)
Supplement: Supplementary file 1 — Supplementary Information [file 41598_2018_20643_MOESM1_ESM.pdf]

Furube et al., (2018) Scientific Reports

Supplementary Figure and Table

Brain Region-dependent Heterogeneity and Dose-Dependent Difference in Transient Microglia Population Increase during Lipopolysaccharide-Induced Inflammation

Eriko Furube, Shintaro Kawai, Haruna Inagaki, Shohei Takagi, Seiji Miyata*

---

*Professor Seiji Miyata,
Department of Applied Biology, Kyoto Institute of Technology, Matsugasaki, Sakyo-ku, Kyoto 606-8585, Japan. smiyata@kit.ac.jp. Tel. and Fax: (+81)75-724-7796

Figure S1

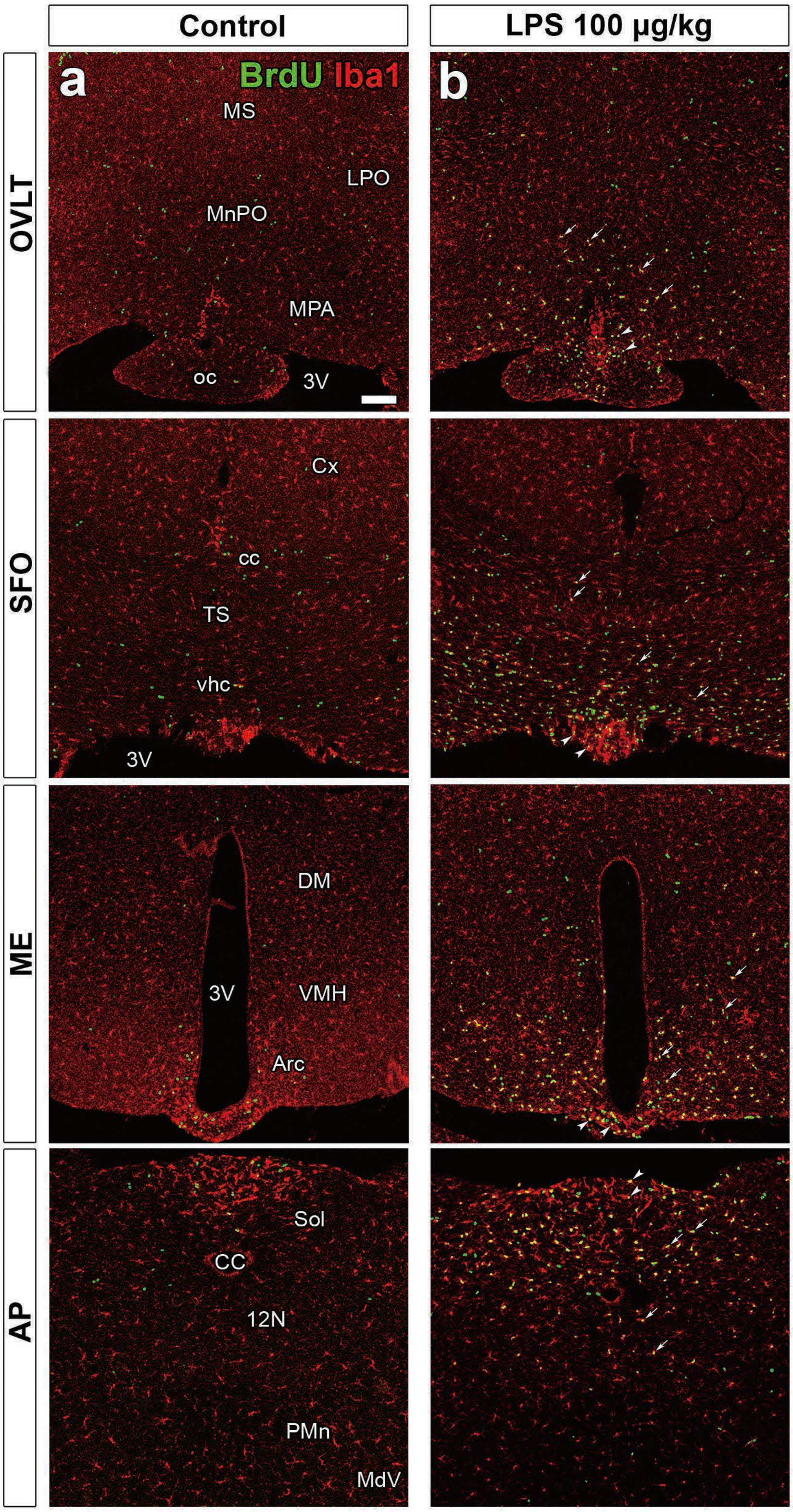

Fig. S1 Low magnification views showing heterogeneous microglial proliferation in the adult mouse brains after the single administration of 100 μg/kg LPS (serotype 055:B5). Animals orally received BrdU via their drinking water (1 mg/ml) for 3 days after the single intraperitoneal administration of LPS. A small number of $BrdU^+$ Iba1-negative cells, presumably neural stem/progenitor cells and endothelial cells, were detected in the CVOs of healthy control mice (**a**). A large number of $BrdU^+$ $Iba1^+$ microglia were observed in the CVOs (arrowheads) and their neighboring brain regions (arrows) after the single intraperitoneal administration of LPS (**b**). Scale bar = 50 μm. DM, dorsomedial hypothalamic nucleus; LPO, lateral preoptic area; MS, medial septal nucleus; oc, optic chiasma; TS, triangular septal nucleus; 3V, 3rd ventricle.

Figure S2

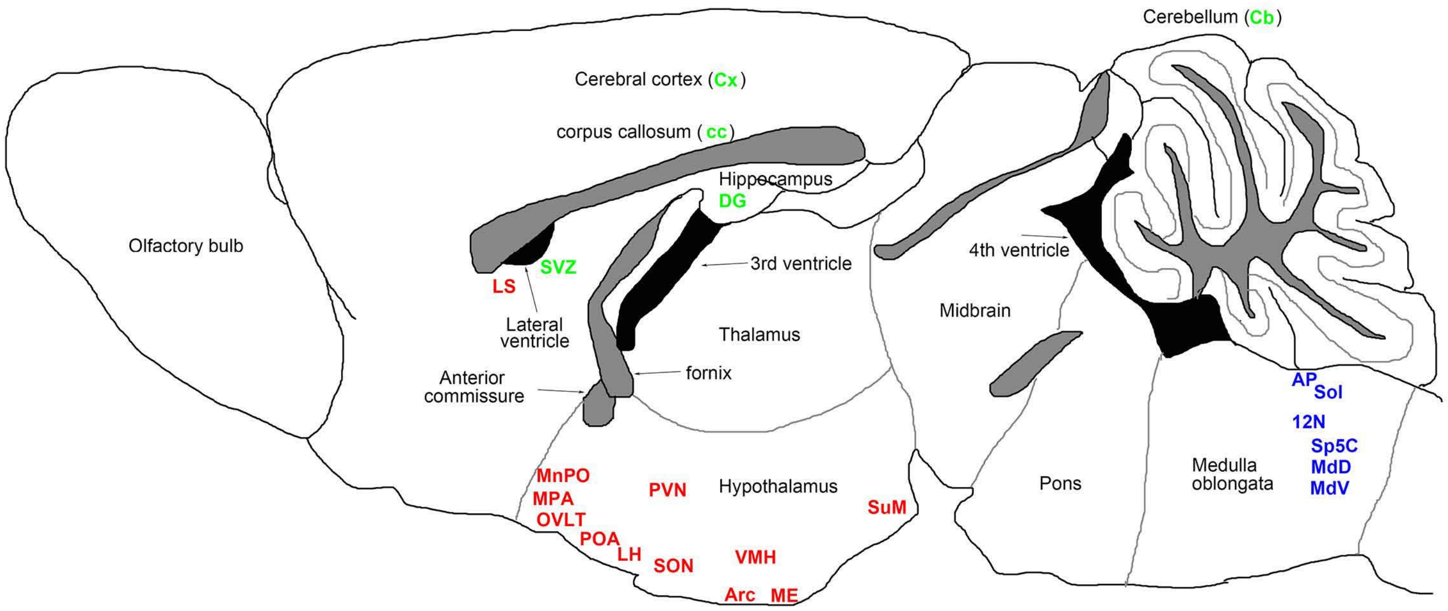

Fig. S2 Schematic representation showing the location of brain regions examined in the present study.

Figure S3

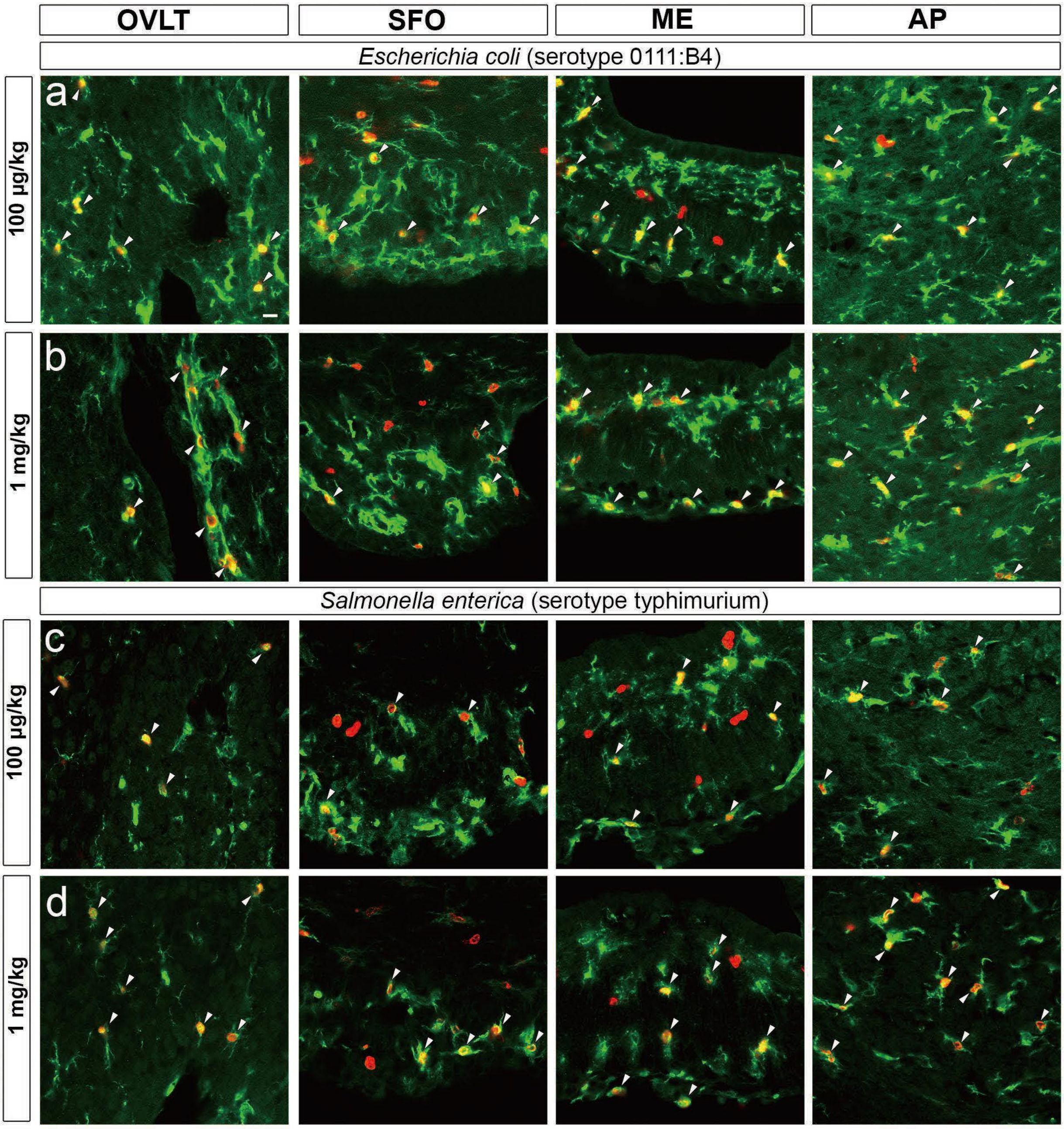

Fig. S3 Prominent increases in the proliferation of microglia/macrophages in the CVOs of the adult mouse after the single intraperitoneal administration of *Escherichia coli* (serotype 0111:B4) and *Salmonella enterica* (serotype typhimurium) LPS. Animals orally received BrdU via their drinking water (1 mg/ml) for 3 days after the single intraperitoneal administration of 100 µg/kg and 1 mg/kg LPS. The single intraperitoneal administration of both B4 (**a,b**) and typhimurium (**c,d**) LPS serotype caused a robust increase in the number of $BrdU^{+}$ and $Iba1^{+}$ microglia/macrophages in the CVOs (arrowheads). Scale bar = 50 µm.

Figure S4

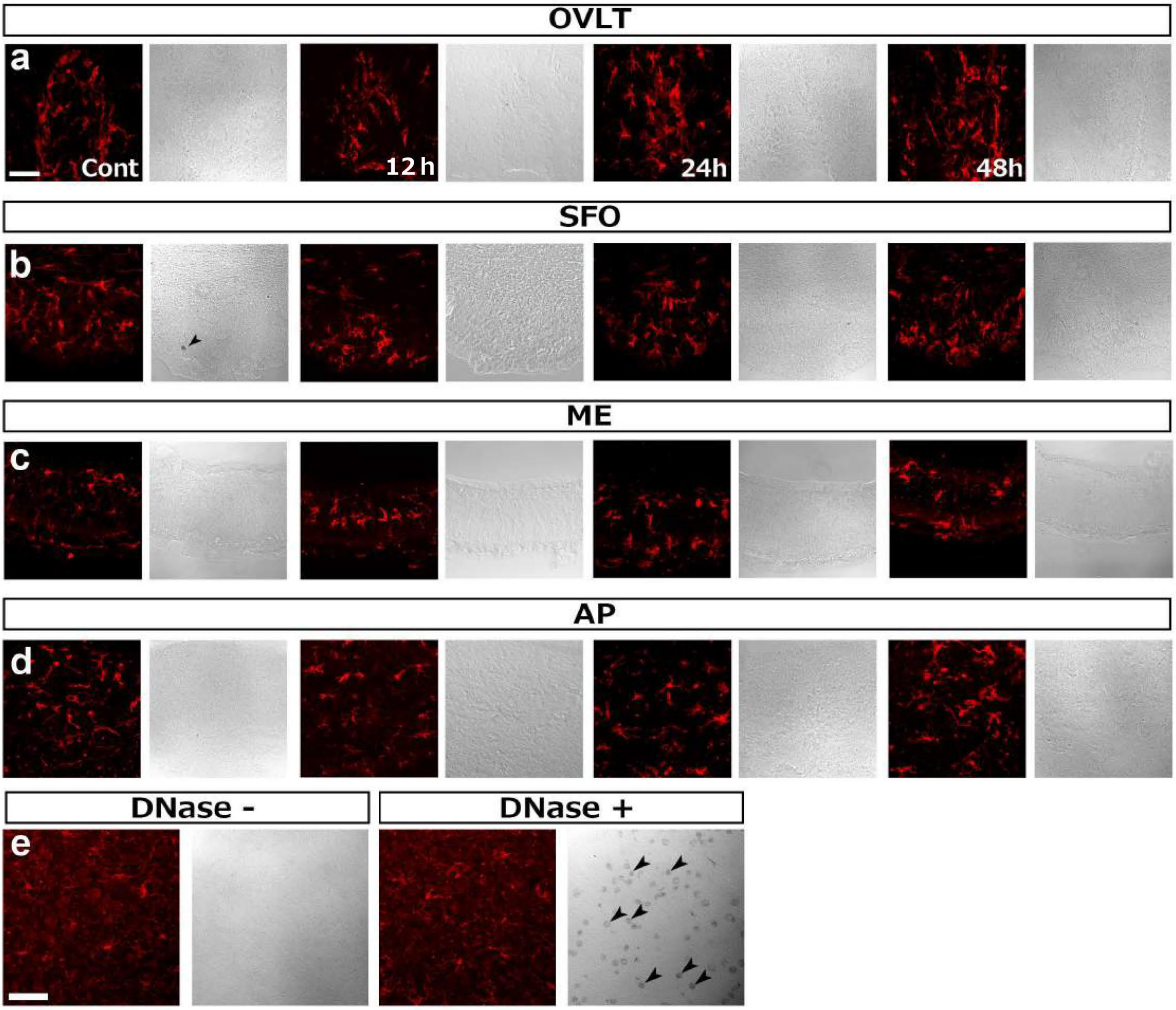

Fig. S4 The TUNNEL assay showing no apoptotic microglia in the CVOs of adult mice after the single administration of 1 mg/kg LPS (serotype 055:B5). Images showed absence of dUTP-incorporated apoptotic microglia at 12, 24, and 48 hr (**a,b,c,d**) after the single administration of LPS. Many microglia were seen in the section of the cerebral cortex which were pretreated with DNase I (**e**). Scale bars = 50 μm.

Figure S5

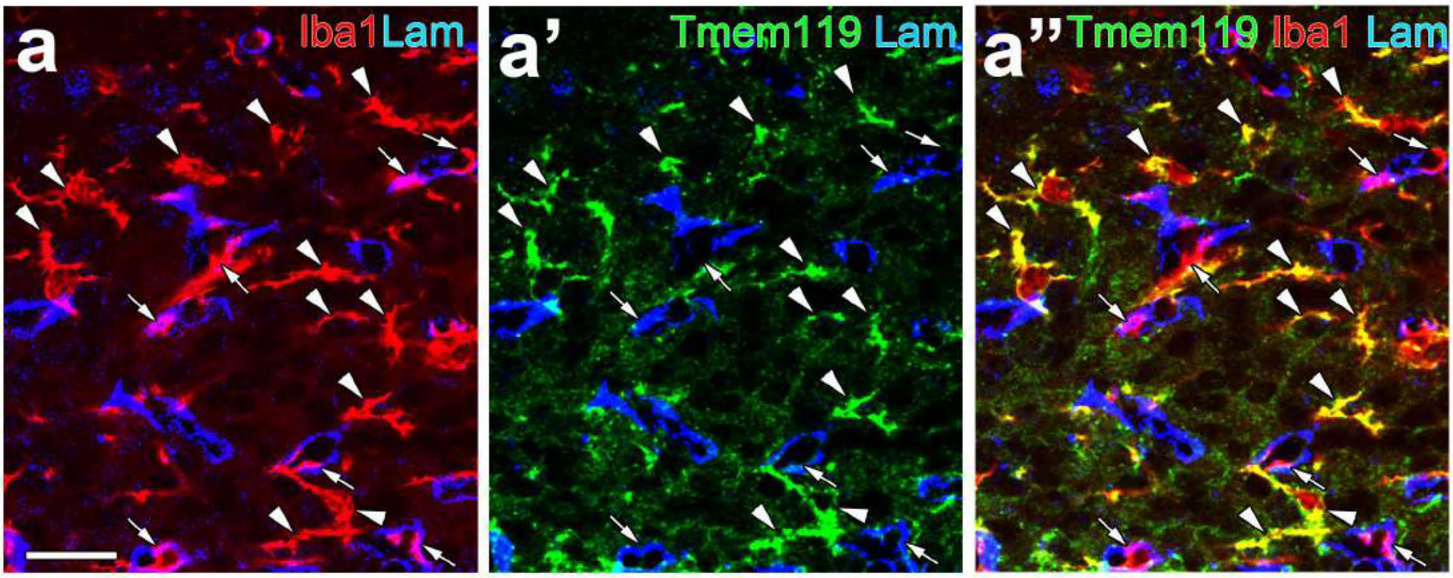

Fig. S5 The immunohistochemistry of Tmem119 showing the specificity of Tmem119 antibody for parenchyma microglia of mouse brains. The immunoreactivity of Tmem119 was seen at Iba1$^+$ microglia (arrows) in brain parenchyma, but it was not observed at Iba1$^+$ macrophages (arrowheads) within laminin-positive perivascular space. Lam, laminin. Scale bar = 50 μm.

Figure S6

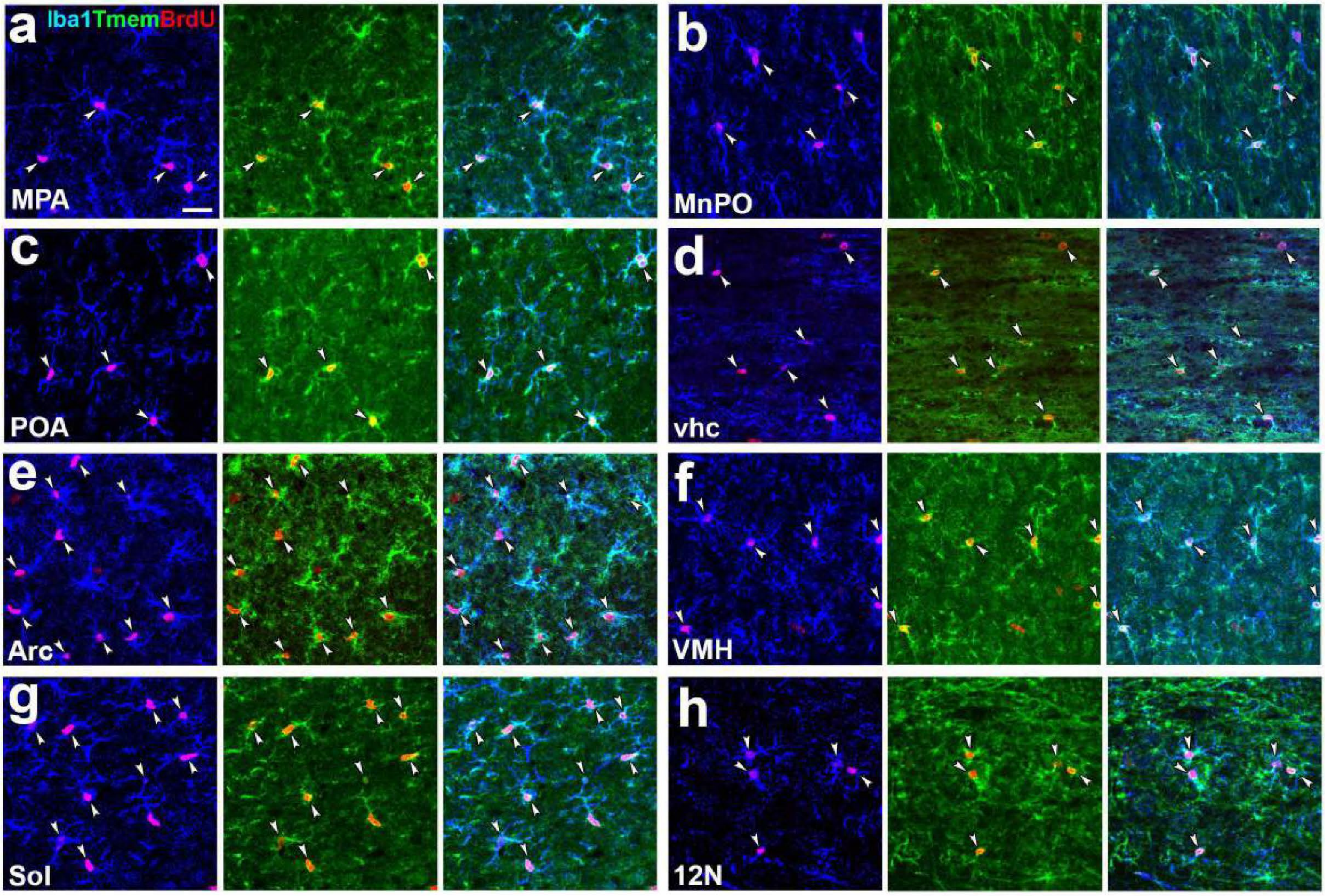

Fig. S6 Demonstration for the proliferation of brain-resident microglia in the brain regions neighboring to the CVOs of the adult mouse after the single administration of 1 mg/kg LPS. Mice orally received BrdU via their drinking water (1 mg/ml) after the single administration of 1 mg/kg LPS (serotype 055:B5) and fixed 3 days later for the immunohistochemistry of Tmem119. Triple labeling immunohistochemistry showed the presence of BrdU^{+} nuclei (arrowheads) in Iba1^{+} and Tmem119^{+} microglia after the single administration of 1 mg/kg LPS. Scale bar = 50 μm.

Figure S7

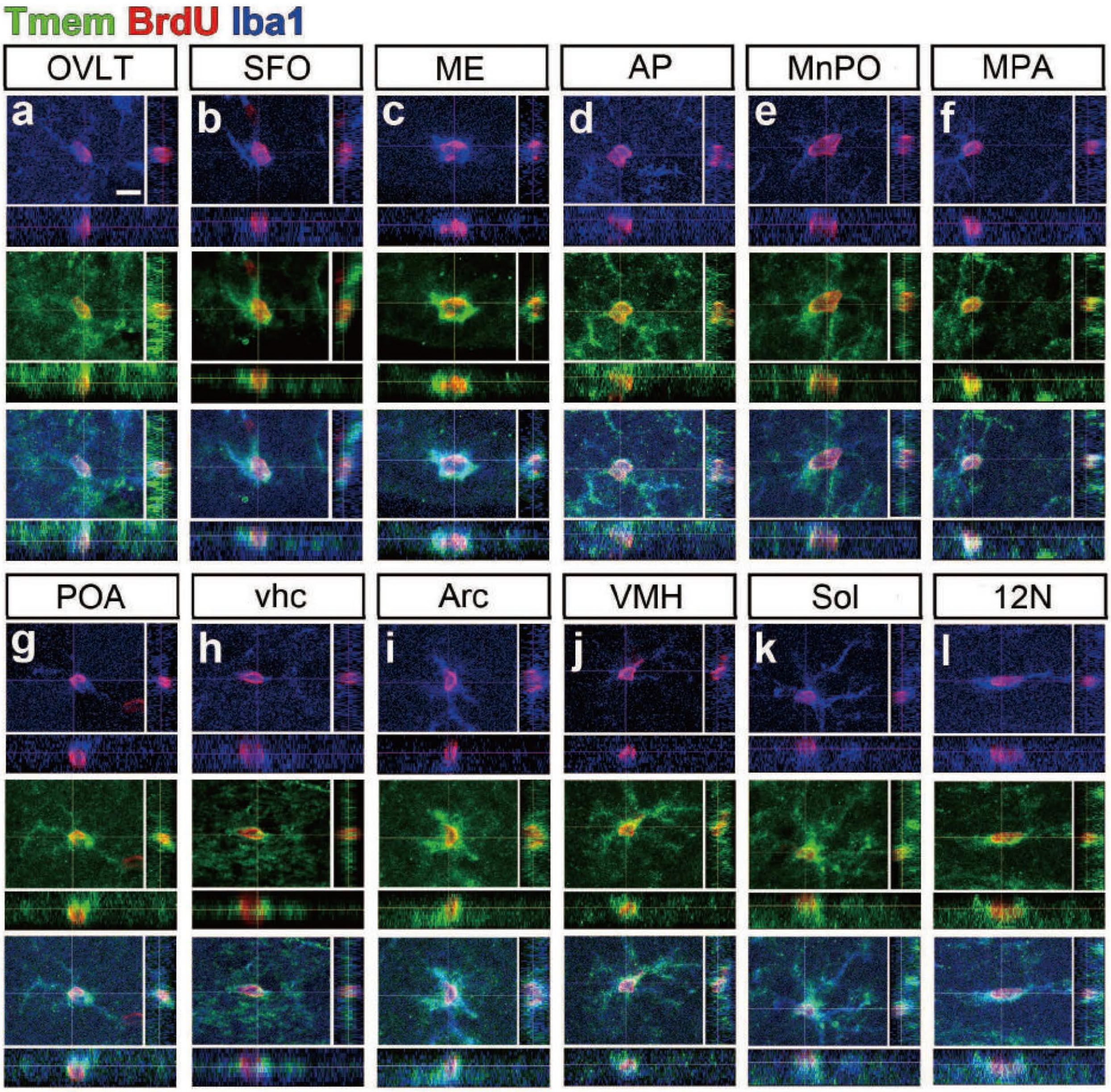

Fig. S7 Three dimensional analysis for the presence of BrdU$^+$ nuclei in Tmem119$^+$ and Iba1$^+$ microglia in the CVOs (**a-d**) and their neighboring brain regions (**e-l**) of the adult mouse brains after the single administration of 100 μg/kg LPS (serotype 055:B5). Animals orally received BrdU via their drinking water (1 mg/ml) for 3 days after the single intraperitoneal administration of LPS and fixed for the immunohistochemistry. Scale bar = 50 μm.

Figure S8

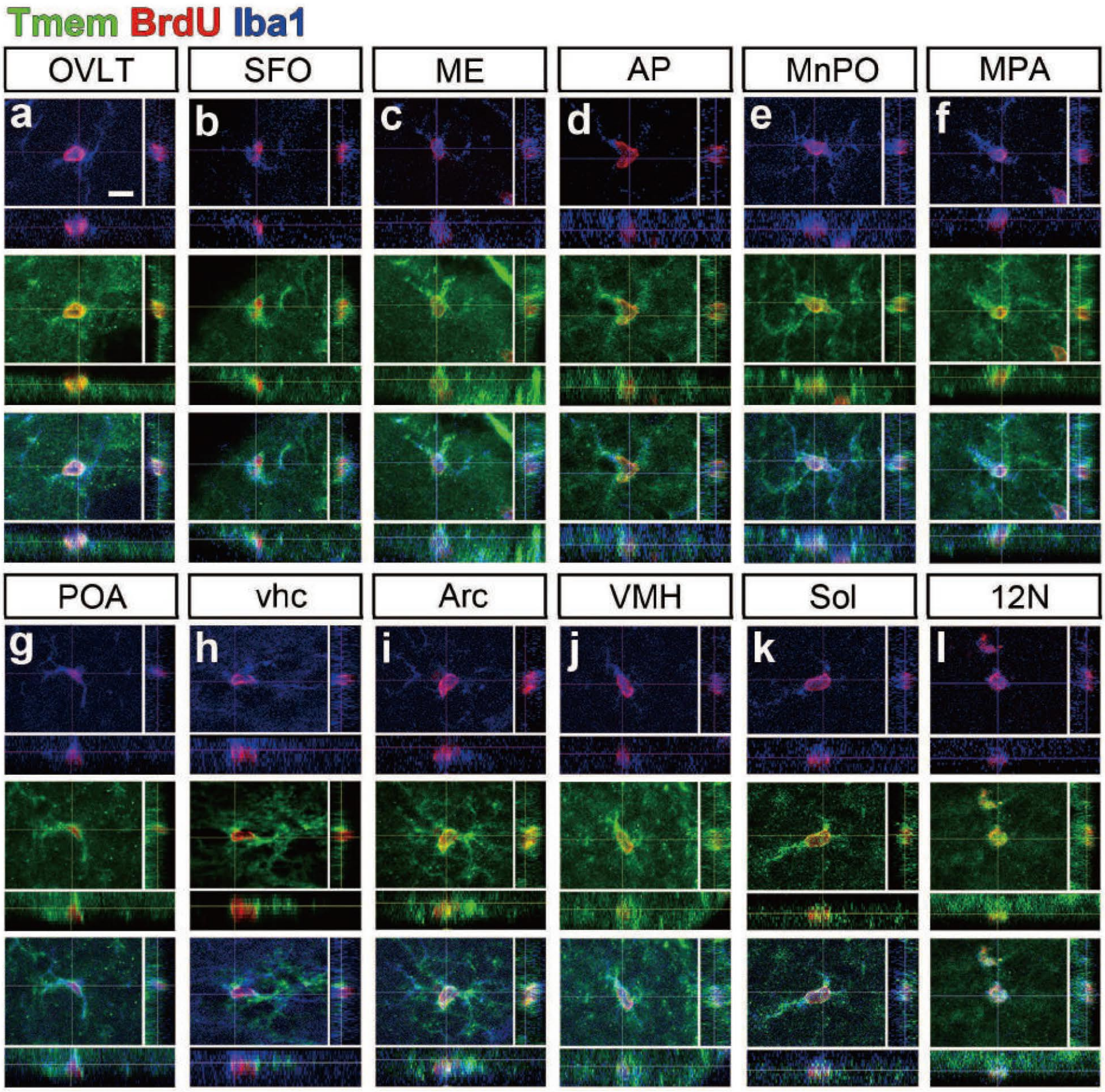

Fig. S8 Three dimensional analysis for the presence of BrdU$^+$ nuclei in Tmem119$^+$ and Iba1$^+$ microglia in the CVOs (a-d) and their neighboring brain regions (e-l) of the adult mouse brains after the single administration of 1 mg/kg LPS (serotype 055:B5). Animals orally received BrdU via their drinking water (1 mg/ml) for 3 days after the single intraperitoneal administration of LPS and fixed for the immunohistochemistry. Scale bar = 50 μm.

Figure S9

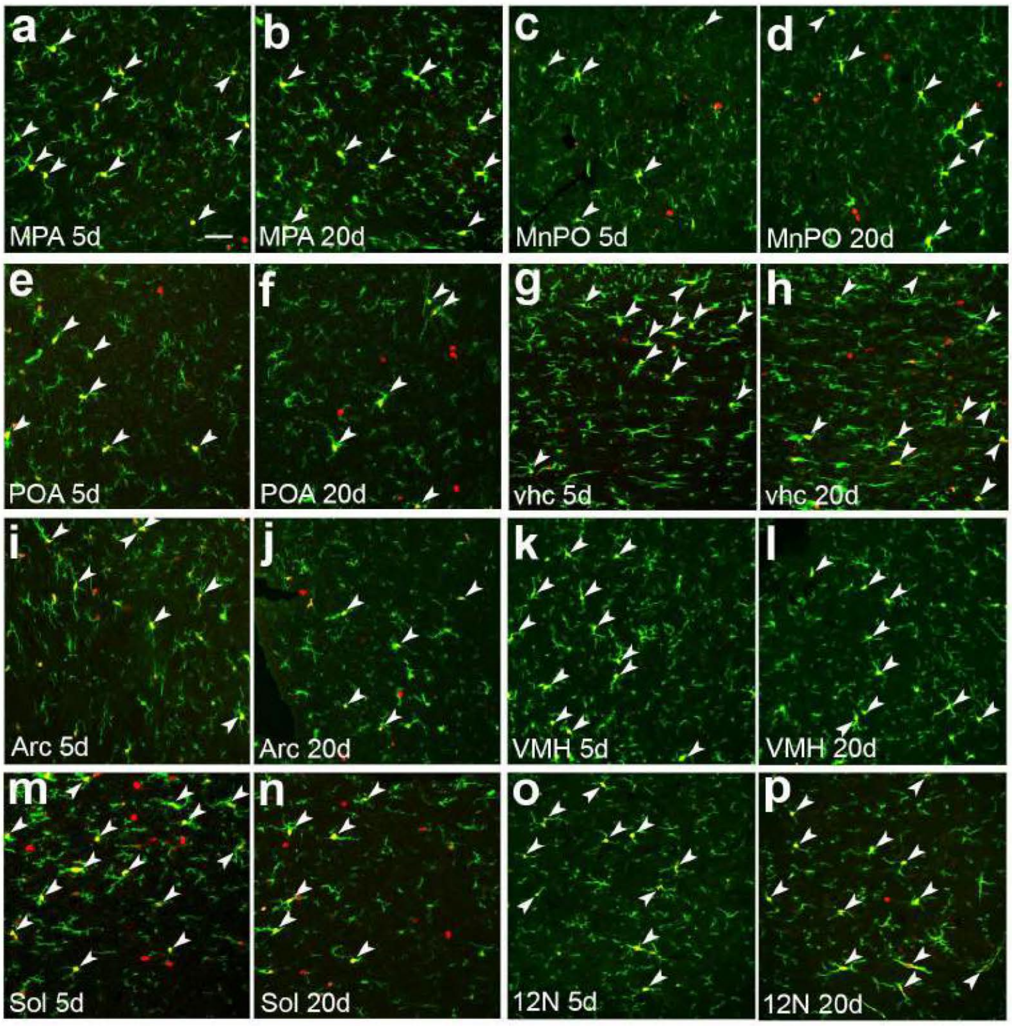

Fig. S9 Survival of BrdU-labeled microglia in the brain regions neighboring to the CVOs in the adult mouse brain on 20th day after the single administration of 100 μg/kg LPS. Animals orally received BrdU via their drinking water (1 mg/ml) for 5 days after the administration of 100 μg/kg LPS (serotype 055:B5) and fixed on 5th and 20th day after the LPS stimulation. Confocal images showed that many BrdU$^+$ and Iba1$^+$ microglia were seen on 20th day after the LPS stimulation as on 5th day. Scale bar = 50 μm.

Figure S10

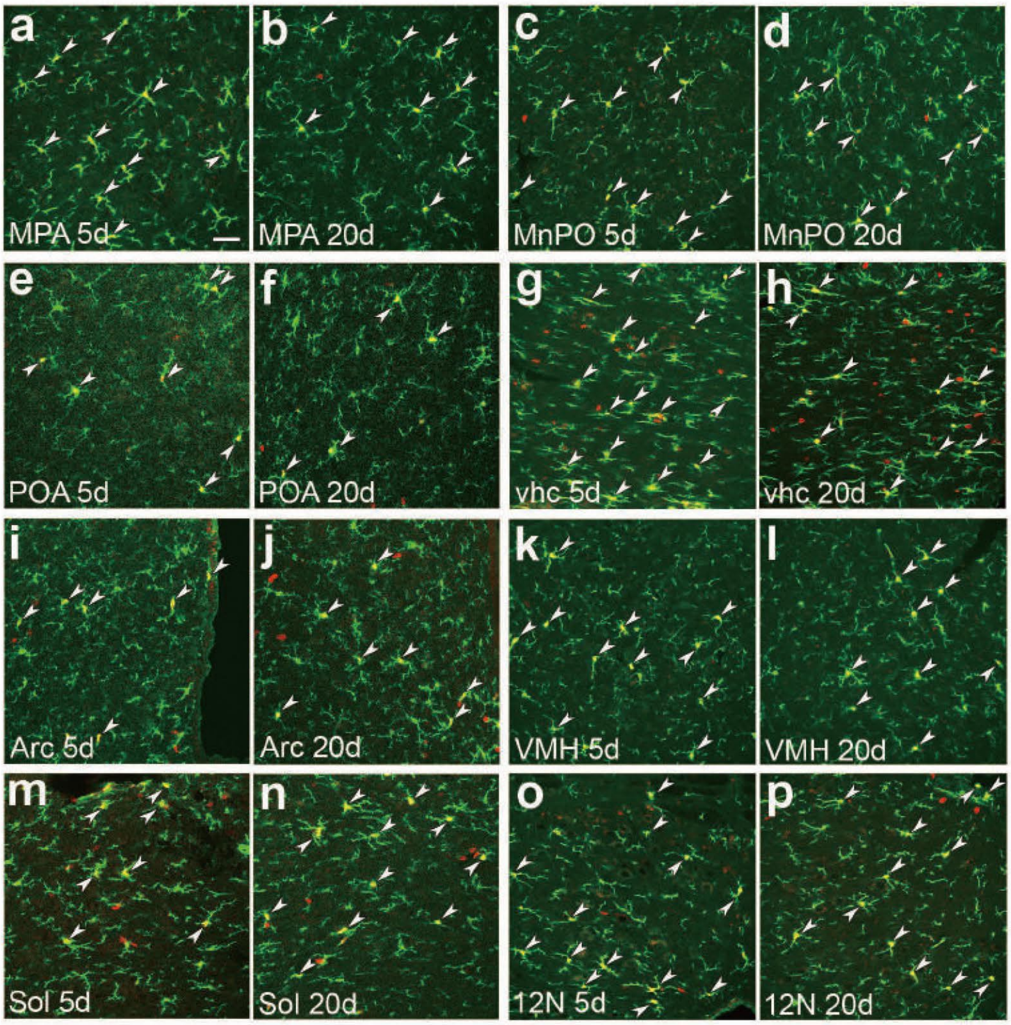

Fig. S10 Survival of BrdU-labeled microglia in the brain regions neighboring to the CVOs in the adult mouse brain on 20th day after the single administration of 1 mg/kg LPS. Animals orally received BrdU via their drinking water (1 mg/ml) for 5 days after the administration of 1 mg/kg LPS (serotype 055:B5) and fixed on 5th and 20th day after the LPS stimulation. Confocal images showed that many BrdU$^+$ and Iba1$^+$ microglia were seen on 20th day after the LPS stimulation as on 5th day. Scale bar = 50 μm.

Supplementary Table 1

**AP**	area postrema
**Arc**	arcuate nucleus
**cc**	corpus callosum
**CVOs**	circumventricular organs
**Cu**	cuneate nucleus
**DG**	dentate gyrus
**LH**	lateral hypothalamic area
**LPO**	lateral preoptic area
**LS**	septal nucleus
**MdD**	dorsal part of the medullary reticular nucleus
**MdV**	ventral part of the medullary reticular nucleus
**ME**	median eminence
**MnPO**	median preoptic nucleus
**MPA**	medial preoptic area
**12N**	hypoglossal nucleus
**OVLT**	the organum of vasculosum of the lamina terminalis
**PMn**	paramedian reticular nucleus
**POA**	preoptic area
**PVN**	paraventricular nucleus
**SFO**	subfornical organ
**Sol**	nucleus of the solitary tract
**SON**	supraoptic nucleus
**Sp5C**	caudal part of the spinal trigeminal nucleus
**SuM**	supramammillary nucleus
**SVZ**	subventricular zone
**vhc**	ventromedial hippocampal commissure
**VMH**	ventromedial hypothalamic nucleus
